# The extracellular matrix protein agrin is essential for epicardial epithelial-to-mesenchymal transition during heart development

**DOI:** 10.1242/dev.197525

**Published:** 2021-05-10

**Authors:** Xin Sun, Sophia Malandraki-Miller, Tahnee Kennedy, Elad Bassat, Konstantinos Klaourakis, Jia Zhao, Elisabetta Gamen, Joaquim Miguel Vieira, Eldad Tzahor, Paul R. Riley

**Affiliations:** 1 Burdon-Sanderson Cardiac Science Centre, Department of Physiology, Anatomy and Genetics, University of Oxford, Oxford OX1 3PT, UK; 2 British Heart Foundation - Oxbridge Centre of Regenerative Medicine, CRM, University of Oxford, Oxford OX1 3PT, UK; 3 Department of Molecular Cell Biology, Weizmann Institute of Science, Rehovot 76100, Israel

**Keywords:** Epicardium, EMT, ECM, Agrin, Dystroglycan, Golgi apparatus, WT1, Mouse

## Abstract

During heart development, epicardial cells residing within the outer layer undergo epithelial-mesenchymal transition (EMT) and migrate into the underlying myocardium to support organ growth and morphogenesis. Disruption of epicardial EMT results in embryonic lethality, yet its regulation is poorly understood. Here, we report epicardial EMT within the mesothelial layer of the mouse embryonic heart at ultra-high resolution using scanning electron microscopy combined with immunofluorescence analyses. We identified morphologically active EMT regions that associated with key components of the extracellular matrix, including the basement membrane-associated proteoglycan agrin. Deletion of agrin resulted in impaired EMT and compromised development of the epicardium, accompanied by downregulation of Wilms’ tumor 1. Agrin enhanced EMT in human embryonic stem cell-derived epicardial-like cells by decreasing β-catenin and promoting pFAK localization at focal adhesions, and promoted the aggregation of dystroglycan within the Golgi apparatus in murine epicardial cells. Loss of agrin resulted in dispersal of dystroglycan *in vivo*, disrupting basement membrane integrity and impairing EMT. Our results provide new insights into the role of the extracellular matrix in heart development and implicate agrin as a crucial regulator of epicardial EMT.

## Abstract (precis)

**Summary:** High-resolution imaging reveals epithelia-to-mesenchyme transition (EMT) heterogeneity on the surface of developing mouse hearts. Proteoglycan again regulates epicardial EMT through dystroglycan localising within the Golgi apparatus.

## INTRODUCTION

The formation and growth of the mouse embryonic heart is a highly dynamic process that includes heart tube elongation and looping, chamber septation and growth. Early specified cardiac progenitors in the linear heart tube rapidly proliferate and differentiate, followed by the incorporation of distinct progenitors from the second heart field to support the structure and function of the mature four-chambered heart (reviewed by Meilhac and Buckingham, 2018; Günthel et al., 2018). Other cell lineages, such as the epicardium, endocardium and neural crest, also contribute fibroblasts, endothelium and vascular smooth muscle to the developing heart (reviewed by von Gise and Pu, 2012; Meilhac and Buckingham, 2018). In addition, proper deposition and modification of the extracellular matrix (ECM) is required for effective attachment, migration and differentiation of the various cardiovascular cell types. However, the ECM has received comparatively less research-focus and, as a consequence, the molecular determinants regulating the interactions between distinct cell compartments and matrix in the embryonic heart remain poorly understood.

ECM is the protein scaffold that not only supports cell attachment but also acts as a reservoir for signaling molecules (reviewed by Hynes, 2009, 2014). The ECM dynamically interacts with cells to regulate their behavior and in turn cells feedback by modifying the matrix to adapt to their changing cell fate and condition the local environment. ECM mainly comprises extensive networks of collagen and laminin, with basement membrane attachments mediated by proteoglycans and glycoproteins (reviewed by Rozario and DeSimone, 2010; Bonnans et al., 2014; Hynes, 2014; Walma and Yamada, 2020). The importance of ECM in various physiological processes has been the focus of a number of previous studies (reviewed by Larsen et al., 2006; Neill et al., 2015). During heart development, the ECM has been implicated in several processes, such as mesendoderm cell fate decisions, trabeculation and valve formation (Cheng et al., 2013; del Monte-Nieto et al., 2018; Gunawan et al., 2019). Within the epicardium, the layer of mesothelial cells covering the outer surface of the myocardium, which is essential for heart development, ECM components have been reported to contribute to tertiary structures within the mesothelium and establish a stem cell-like niche (Balmer et al., 2014); however, besides maintaining structural integrity, regulatory roles for the ECM within the epicardium and more generally during heart development have not been studied to date.

In mouse, the epicardium is derived from the proepicardium (PE), which is a transient embryonic structure attached at the base of the venous inflow tract. At embryonic day (E) 9.5, PE cells migrate to cover the surface of the myocardium forming an outer cell layer by E11.5. A subset of epicardial cells undergo epithelial-to-mesenchymal transition (EMT) and invade the underlying myocardium, with epicardium-derived cells (EPDCs) then differentiating into multiple cardiovascular cell types, including fibroblasts and vascular smooth muscle cells, to support coronary vessel development and growth of the embryonic heart (reviewed by Riley, 2012; Simões and Riley, 2018; Cao and Poss, 2018). Disruption of epicardial development leads to mid-gestation lethality, with mutant mice exhibiting underdeveloped hearts (Yang et al., 1995; Kwee et al., 1995). For instance, loss of the transcription factor Wilms’ tumor 1 (WT1), a master epicardial cell regulator, resulted in defective epicardial EMT, hypoplastic myocardium and embryonic death (Moore et al., 1999; Zhou et al., 2008; von Gise et al., 2011). WT1, along with several other factors such as fibroblast growth factor (FGF), transforming growth factor beta (TGFβ) and thymosin beta 4 (Tβ4), has been shown to be crucial for the formation of the epicardium, maintenance of structural integrity of the epithelium, and epicardial EMT, suggesting possible roles for the ECM of the basement membrane of the epicardium (Vega-Hernández et al., 2011; Compton et al., 2007; Smart et al., 2007). However, the precise spatial-temporal events supporting epicardial EMT have not been described in detail. Moreover, how ECM and intracellular signals interact and enable mesenchymal transition and epicardial cell migration is unknown.

Agrin is an important component of the ECM with signal-mediating potential that ensures connectivity between cells and the basement membrane. It is a heparan-sulphate proteoglycan (HSPG) broadly expressed in muscle, neuron, blood and endothelial cells (Stone and Nikolics, 1995). Agrin was first identified as a protein that can promote the aggregation of acetylcholine receptors on the muscle-neuron surface through muscle-synapse kinase (MuSK) (Nitkin et al., 1987; Gautam et al., 1996; Lin et al., 2001). More recently, it has been implicated in promoting cancer progression and invasion (Chakraborty et al., 2015; Rivera et al., 2018). In the brain endothelium, agrin deposits in the basement membrane, ensheathing blood vessels and contributing to blood-brain barrier maturation (Steiner et al., 2014). Agrin has also been implicated as an important contributor to cardiomyocyte turnover in the context of heart regeneration and repair in both mice and pigs (Bassat et al., 2017; Baehr et al., 2020; Bigotti et al., 2020). Mechanistically, agrin transduces tissue rigidity signals and stabilizes the Yes-associated protein (YAP; also known as YAP1) (Chakraborty et al., 2017) target pathways implicated in inducing cardiomyocyte cell cycle re-entry (Bassat et al., 2017; Baehr et al., 2020). However, the role of agrin in development per se and more specifically within the embryonic heart has not been characterized.

Here, we describe for the first time a requirement for agrin in the embryonic mouse heart as an integral link between the ECM and epicardial development. We employed high-resolution imaging using combined scanning electron microscopy (SEM) and confocal microscopy to reveal that the embryonic epicardium comprises a highly heterogenous morphology with evidence of epicardial cells undergoing regionalized EMT. The ECM components laminin and integrin α4, agrin and its receptor dystroglycan were associated with active regions of EMT in the developing epicardium. Loss of agrin resulted in pleiotropic defects, including abnormal deposition of epicardial ECM and decreased EMT of epicardial cells. Exogenous agrin promoted EMT and activated the integrin-focal adhesion kinase (FAK) signaling pathway in a model of human embryonic stem cell-derived epicardial cells. Additionally, we identified dystroglycan aggregation to Golgi apparatus as the signal transduction pathway for agrin in the epicardial cells and a requirement for maintaining basement membrane and cytoskeletal connectivity during EMT. Taken together, our study identifies the ECM component agrin as a novel determinant of normal heart development and a crucial regulator of epicardial EMT.

## RESULTS

### Epicardial cells with distinct morphology and EMT status are present on the surface of the embryonic heart

**Fig. 1.**
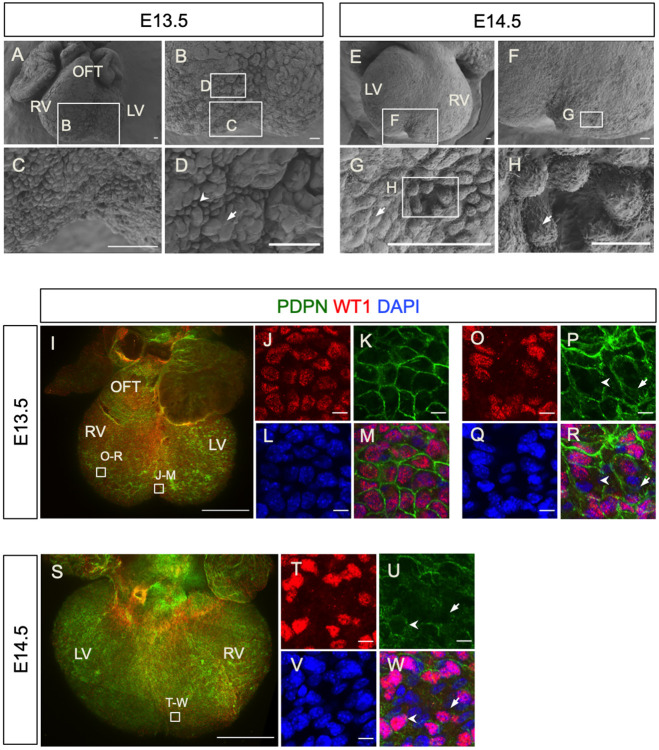
**The developing epicardium has active regions of EMT and exhibits morphological heterogeneity.** (A-H) The surfaces of E13.5 and E14.5 hearts viewed by SEM. (A) The ventral aspect of an E13.5 heart. (B) Magnified view of the boxed area in A. (C,D): Magnified views of the boxed areas in B. (E) The dorsal aspect of an E14.5 heart. (F) Magnified view of the boxed area in E. (G) Magnified view of the boxed area in F. (H) Magnified view of the boxed area in G. Note the distinct cell morphologies, including flat and tightly connected cells (arrow in D,G) and cells detached from each other suggesting undergoing EMT (arrow in D,H). (I-R) Whole-mount immunofluorescence staining for the epicardium markers WT1 (red) and podoplanin (PDPN, green) and DAPI (blue) in an E13.5 heart (ventral aspect). (J-M) Magnified view of the boxed area in I at the ventricular septation. (O-R) Magnified view of the boxed area in I on the ventricle wall. (S-W) Whole-mount immunofluorescence staining for WT1 (red), podoplanin (green) and DAPI (blue) in an E14.5 heart (dorsal aspect). (T-W) Magnified view of the boxed area in S near the ventricle septum. LV, left ventricle; OFT, outflow tract; RV, right ventricle. Scale bars: 50 μm (A-G); 10 μm (H); 500 μm (I,S); 10 μm (J-M,O-R,T-W).

To investigate changes in cell morphology during epicardial EMT, we initially examined the outer surface of the developing mouse heart at E13.5 and E14.5 by SEM (Fig. 1A-H). At E13.5, the ventral surface of the heart was found to be irregular (Fig. 1A, *n*=5), with clusters of rosette-like structures separated by grooves being observed at higher magnification (Fig. 1B-D). By comparison, the dorsal epicardial surface appeared to be smoother (Fig. S1A), and two distinct cell morphologies could be observed on the epicardial surface: large flat-shaped cells connecting with neighboring cells with well-defined cell-cell borders (Fig. S1B, arrow) and loosely connected small pillar-shaped cells (Fig. S1B,C, arrowheads). The latter morphology type was represented throughout the heart surface, with small clusters (Fig. 1D, arrow) being observed among patches of tightly connected, large flat cells (Fig. 1D, arrowhead). These two cell morphologies were also observed on the epicardial surface at E14.5 (Fig. 1E-H, *n*=6). The large flat surface cells exhibited finger-shaped protrusions mainly along the cell-cell borders (Fig. 1G, arrow; Fig. S1B, arrow), whereas such protrusions were distributed on the apical and lateral sides of small pillar-shaped surface cells (Fig. 1H, arrow; Fig. S1C). Importantly, the changes in cell morphology observed are consistent with the definition of cells undergoing EMT, i.e. transition from a large flat shape into a small pillar-like structure (Yang et al., 2020).

To characterize further the epicardial surface during heart development, we performed whole-mount immunofluorescence staining using antibodies against the well-established epicardial markers podoplanin (PDPN) and WT1 (Mahtab et al., 2008; Moore et al., 1999; Zhou et al., 2008). PDPN immunoreactivity was detected on the lateral borders of epicardial cells, whereas WT1 expression was restricted to their nuclei (Fig. 1I-W). We observed large, round WT1^+^ nuclei with PDPN^+^ cell borders near the ventricular septum (Fig. 1J-M) and epicardial cells undergoing EMT within the epithelium in this region (Fig. 1F,G). On the ventricular wall, the WT1^+^ nuclei varied in shape as round, oval or rod-like. Some of the cells in the epicardium had downregulated WT1 or lacked WT1 in their nuclei, although PDPN was detected in their membranes (Fig. 1O,P,R, arrows), suggesting they were differentiating epicardial cells. Interestingly, we observed small regions with multiple WT1^+^ and WT1^−^ nuclei, but no PDPN^+^ cell borders separating them (Fig. 1P,R, arrowheads). We hypothesized that absence of podoplanin in these regions may be associated with EMT regions where cell-cell connections are lost, as observed in our SEM analyses (Fig. S1B,C). At E14.5, we observed similar epicardial cell morphologies (Fig. 1S-W). WT1^+^ epicardial cells enclosed with defined PDPN^+^ borders were evident (Fig. 1U,W, arrowheads). alongside regions in which PDPN expression was low and discontinuous with both WT1^+^ and WT1^−^ nuclei (Fig. 1U,W, arrows) and, as for E13.5, potentially labeling active regions of epicardial EMT. On the outflow tract, SEM revealed that epicardial cells were small and round and tightly packed together (Fig. S2A). Whole-mount immunofluorescence staining confirmed the cells at this location were WT1 and PDPN positive (Fig. S2B-D). In contrast, cells at the junction of the outflow tract with the chambers were large and flat (Fig. S2A). These cell shape differences were also observed by immunofluorescence staining for PDPN (Fig. S2C). In summary, combined SEM and immunofluorescence staining revealed regional and heterogeneous cell morphology within the developing epicardium, which has not previously been described and which we attribute to the EMT status of the constituent epicardial cells.

To validate the EMT heterogeneity of epicardial cells, we investigated expression of the EMT markers ZEB1 and ZO1 (also known as TJP1), respectively. ZEB1 is a zinc-finger transcription factor reported to induce EMT by suppressing epithelial genes (Thiery et al., 2009; Brabletz and Brabletz, 2010; Lamouille et al., 2014). ZO1 is an important component of cell-cell tight junctions (reviewed by Zahraoui et al., 2000). In sections of E13.5 and E14.5 hearts (Fig. S3A,D), we identified PDPN^+^ cells expressing both high nuclear ZEB1 (Fig. S3B,E, arrows) and low nuclear ZEB1 (Fig. S3C,F, arrows), suggesting different EMT potential of these cells. Similarly, we also observed PDPN^+^ cells with low ZO1 (Fig. S3H,I, arrows) in E13.5 and E14.5 hearts (Fig. S3G,J), indicating reduced tight cell junctions as a precursor for EMT and PDPN^+^ cells with high ZO1 expression (Fig. S3I,L, arrows), indicative of their epithelial status. The heterogeneity of ZEB1 and ZO1 expression suggests that epicardial cells on the surface of the embryonic heart exist in different EMT states, consistent with the morphological observations by SEM.

### Active epicardial EMT regions have a distinct ECM composition

As EMT is associated with dynamic basement membrane breakdown and establishment, we investigated the distribution of key ECM components and integrins, including integrins α4 and β1, laminin and fibronectin, in the developing epicardium, using whole-mount immunofluorescence staining. Integrin α4 is an epicardial-specific integrin subunit (Yang et al., 1995; Kirschner et al., 2006) and integrin β1 is the beta-subunit predominantly expressed in the developing heart (Ieda et al., 2009). Laminin is a major component of the basal membrane (reviewed by Yurchenco, 2015). Fibronectin is a high-molecular-weight glycoprotein that serves as a receptor for various integrins (reviewed by Pankov and Yamada, 2002) and is secreted by epicardial cells and necessary for heart regeneration in zebrafish (Wang et al., 2013). These proteins were all detected on the surface of the embryonic heart at E14.5 (Fig. S4A-D), with closer examination showing enrichment in regions proximal to the forming coronary vasculature (Fig. S4E-H).

**Fig. 2.**
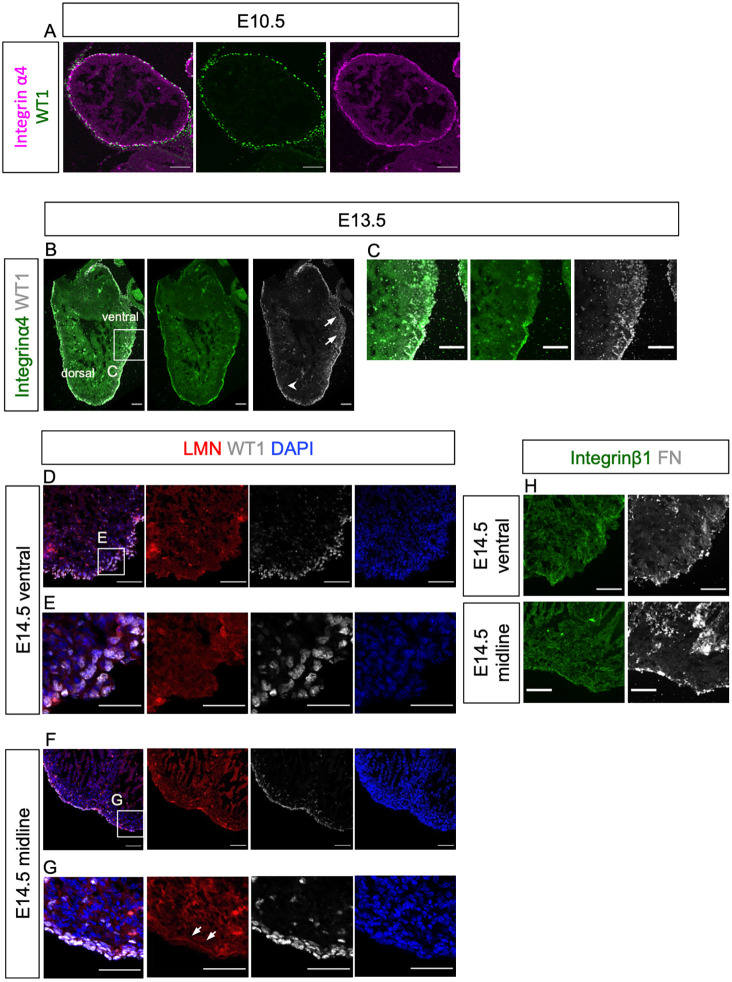
**Active EMT regions within the embryonic epicardium are associated with ECM components.** (A) Immunofluorescence staining of a sagittal section of an E10.5 heart for WT1 (green) and integrin α4 (magenta). (B,C) Immunofluorescence staining of a sagittal section of an E13.5 heart for WT1 (white), integrin α4 (green) and DAPI (blue) showing clusters of WT1^+^ cells migrating into the myocardium and downregulated integrin α4 around these cells (arrows in B), and WT1^+^ cells localizing within the epicardium (arrowhead in B) (C) Magnified view of the boxed area in B. (D-G) Immunofluorescence staining for laminin (LMN; red), WT1 (white) and DAPI (blue) on serial sections from an E14.5 heart, near the ventral surface (D,E) and near the dorsal-ventral midline (F,G). (E) Magnified view of the boxed area in D. (G) Magnified view of the boxed area in F. Arrowheads in G indicate continuous LMN in basal membrane. (H) Immunofluorescence staining of an adjacent section to that shown in D (upper panels) and to that shown in F (lower panels) for integrin β1 (green) and fibronectin (FN; white). Scale bars: 50 μm (A); 100 μm (B,C); 50 μm (D-H).

To investigate further the association of ECM components with epicardial cell delamination and migration into the subepicardial space and underlying myocardium, we analyzed serial sagittal sections of embryonic hearts at E10.5, E13.5 and E14.5. The antibody specificity of integrin α4 was validated with immunofluorescence staining of a section of an E10.5 embryo showing signals in the neural tube (Stepp et al., 1994) and somites (Bajanca et al., 2004) (Fig. S5A) and laminin was validated by positive staining of the basal membrane of a blood vasculature (reviewed by Yurchenco, 2015) (Fig. S5B). At E10.5, WT1^+^ cells were encircled by integrin α4 and aligned in a single layer on the surface of the heart (Fig. 2A). At E13.5, WT1^+^ cells constituted multiple-layers on the epicardium at several sites (Fig. 2B). We focused on a region in the ventral side with clusters of WT1^+^ cells migrating from the epicardium to the myocardium (Fig. 2B,C). In this region, WT1^+^ cells with large and round nuclei were found in the underlying myocardium (Fig. 2B,C arrows). Here, integrin α4 expression was weak and discontinuous around migrating WT1^+^ cells (Fig. 2B,C). By contrast, the integrin α4 signal was clearly visible and continuous on the epicardium in other regions of the same sections, where WT1^+^ cells were mainly localized within the epicardium (Fig. 2B, arrowhead). Comparatively fewer WT1^+^ cells were found migrating into the myocardium in these integrin α4-enriched epicardial regions (Fig. 2B). This observation suggests that downregulation of integrin α4 is associated with active EMT.

Examination of a series of long-axis serial sections from hearts at E14.5 revealed active EMT regions (Fig. 2D,E) to be proximal to the ventral surface, whereas low EMT regions (Fig. 2F,G) tended to be distal, closer to the heart midline. Importantly, in the active EMT regions, deposition of laminin around WT1^+^ cells was low and uneven (Fig. 2D,E, red). In this region, the majority of WT1^+^ nuclei were large and round in shape (Fig. 2D,E, white). By contrast, in low EMT regions, laminin formed a continuous sheet on the side of epicardial cells contacting the myocardium (Fig. 2F,G, arrows). WT1^+^ nuclei were rod-shaped and tightly attached within the laminin-marked basal membrane and fewer WT1^+^ cells were detected in the adjacent myocardium (Fig. 2F,G, white). Integrin β1 expression was detected in the epicardium and myocardium (Fig. 2H), with no obvious difference in the high (Fig. 2H, upper green panel) versus low (Fig. 2H, lower green panel) epicardial EMT regions. Likewise, the punctate expression pattern of epicardial fibronectin showed no difference in high- versus low-EMT regions (Fig. 2H, white), suggesting that the distribution of integrin β1 and fibronectin is not associated with epicardial EMT.

### Agrin is expressed in the epicardium and regulates epicardial EMT in the developing heart

**Fig. 3.**
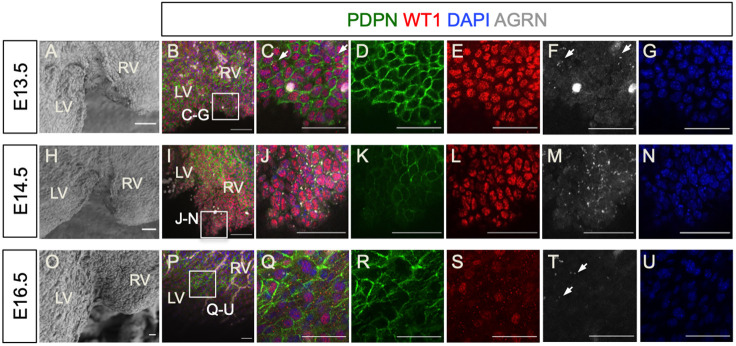
**Localization of agrin in the developing epicardium.** (A,H,O) SEM showing the area near the interventricular septum of E13.5, E14.5 and E16.5 hearts, respectively. (B-G,I-N,P-U) Whole-mount immunofluorescence staining of E13.5, E14.5 and E16.5 heart for podoplanin (PDPN, green), WT1 (red), agrin (white) and DAPI (blue). (C,J,Q) Magnified views of the boxed areas in B,I,P. Agrin localizes among epicardial cells in close proximity to podoplanin (C,F, arrows). The punctate localization of agrin increased at E14.5 (J,M) and decreased at E16.5 (Q,T, arrows). LV, left ventricle; RV, right ventricle. Scale bars: 50 μm.

To characterize further the ECM components associated with epicardial EMT, we next focused on agrin, an important component of the basement membrane that can be bound to the cell membrane or secreted (reviewed by Bezakova and Ruegg, 2003). Using whole-mount immunofluorescence staining, we investigated the expression of agrin in the developing epicardium from E13.5 to E16.5, focusing on regions proximal to the interventricular septum (Fig. 3A,H,O), which appeared enriched for cells undergoing EMT as detected by SEM (Fig. 1). At E13.5, agrin was detected in the WT1^+^ epicardial layer in a sparse, punctate pattern along the cell borders (Fig. 3B-F, arrows), colocalizing with PDPN (Fig. 3C,F). At E14.5, among the tightly packed WT1^+^ PDPN^+^ cells near the septum (Fig. 3I-K), agrin-expressing puncta were more dense (Fig. 3J,M). By E16.5, many epicardial cells had prominently downregulated or lost WT1 expression coincident with completion of EMT and subsequent differentiation (Fig. 3P,Q,S). Likewise, the punctate expression pattern of agrin in epicardial cells was also reduced (Fig. 3T, arrows), suggesting that agrin is associated with epicardial EMT.

**Fig. 4.**
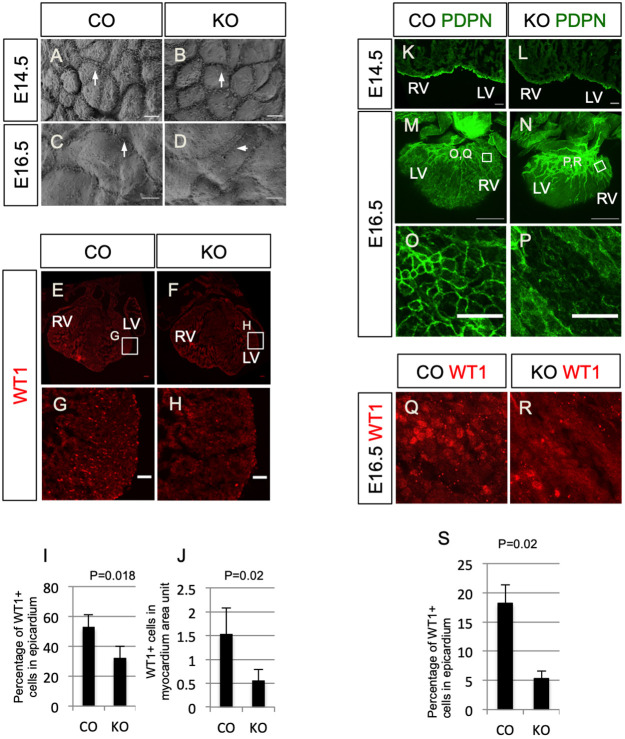
**Loss of agrin results in developmental defects specifically in the epicardium.** (A-D) Epicardial cells on the surface of E14.5 (A) and E16.5 (C) littermate controls (CO) and agrin KO hearts (B,D) hearts under SEM. Arrows in A-D indicate protrusions along cell-cell borders. (E,F) Immunofluorescence staining for WT1 (red) in E14.5 heart sections of the littermate control and agrin KO. (G,H) Magnified views of the boxed areas in E and F. (I) Quantification of WT1^+^ cells in the epicardium of control and KO hearts (percentage of WT1^+^ nuclei among epicardial cells). (J) Quantification of the number of WT1^+^ cells in a designated myocardium area. Data represent mean±s.e.m. *n*=3 hearts per group. (K,L) Immunofluorescence staining for podoplanin (PDPN, green) in E14.5 control (K) and agrin KO (L) sections. (M,N) Whole-mount immunofluorescence staining of E16.5 control (M) and agrin KO (N) hearts for podoplanin (green) labeling cardiac lymphatic vessels and epicardial cells. (O,P) Magnified views of the boxed areas in M and N. Note the downregulated epicardial podoplanin in agrin KO hearts. (Q,R) Whole-mount immunofluorescence staining for WT1 (red) of the same hearts shown in M and N at the same area as shown in O and P. (S) Quantification of the percentage of WT1^+^ cells in epicardium of E16.5 controls and agrin KO. Data represent mean±s.e.m. *n*=3 hearts per group. *P*-value was calculated using an unpaired, two-tailed Student's *t*-test. LV, left ventricle; RV, right ventricle. Scale bars: 5 μm (A-D); 50 μm (E-H,K,L,O,P); 500 μm (M,N).

Given that a requirement for agrin during heart development has not been previously described, we generated global agrin knockout mutants by crossing *Agrn*^flox/flox^ (Harvey et al., 2007) with *Pgk-Cre* mice (Lallemand et al., 1998). The resulting *Agrn*^+/−^ mice were intercrossed to generate *Agrn*^−/−^ embryos, henceforth referred to as agrin KO. Global agrin KO has been confirmed and phenotypes of the resulting mice, not related to the developing heart, have been characterized previously (Gautam et al., 1996; Harvey et al., 2007). SEM was used to initially characterize the epicardial surface of agrin KOs versus control littermate hearts at E14.5 and E16.5. Whereas wild-type epicardial cells displayed multiple protrusions along the cell-cell border and on the cell surface, the density of protrusions appeared reduced on the epicardium of agrin KOs (Fig. 4A,B, arrows). This phenotype was also observed in hearts at E16.5 (Fig. 4C,D, arrows). Fluorescence immunostaining against WT1 revealed that there were fewer WT1^+^ cells on the epicardium as well as in the myocardium [Fig. 4E-J; epicardium: 52.9%±8.27% WT1^+^ cells in wild type versus 32.5%±7.47% WT1^+^ cells in agrin KO, mean±s.e.m., *n*=3, *P*=0.018; myocardium: 1.53±0.55 WT1^+^ cells per artificial unit (1×10^4^ DAPI^+^ pixels) in wild type versus 0.55±0.24 WT1^+^ cells per artificial unit in agrin KO, *n*=3, *P*=0.02], suggesting that formation of the epicardium and epicardial cell migration are agrin dependent. To validate these results, we investigated PDPN immunoreactivity in agrin KO versus control littermates, and found that expression levels were low and discontinuous in the epicardium of mutant hearts at E14.5 (compare Fig. 4K and 4L). Likewise, at E16.5 whole-mount immunofluorescence staining revealed that PDPN expression was downregulated on the epicardial surface of agrin KO hearts (Fig. 4M-P), with the epicardial cell-cell border barely discernible (compare Fig. 4O with 4P). Furthermore, the growth and patterning of PDPN-expressing lymphatic vessels in the subepicardial layer appeared defective in KO versus wild-type hearts (compare Fig. 4M with 4N). The expression of WT1 was also decreased in the outer surface of KO hearts at E16.5 (Fig. 4Q,R; note that these panels are from the same region as in Fig. 4O and 4P). At this stage, 18.17±3.21% of cells in the epicardium of controls expressed WT1^+^, whereas in the agrin KO only 5.29%±1.36% of cells were WT1^+^ (*n*=3, *P*=0.02; Fig. 4S). In summary, loss of agrin resulted in abnormal epicardial development and impaired EMT.

Because the disruption of epicardial development may affect the formation of coronary vasculature (von Gise et al., 2011), we investigated whether loss of agrin also resulted in vascular defects. At E14.5, agrin KOs exhibited shortening of the superficial coronary veins as depicted by endomucin (EMCN) staining in the dorsal aspect of the heart (Fig. S6A,B, arrows). This defect was also observed in hearts at E16.5 (Fig. S6C,D, arrows) and by E18.5 the coronary vessels in agrin KO hearts appeared to have lost vessel integrity with PECAM^+^ endothelial cells failing to form a fully enclosed lumen (Fig. S6E-H, arrow).

**Fig. 5.**
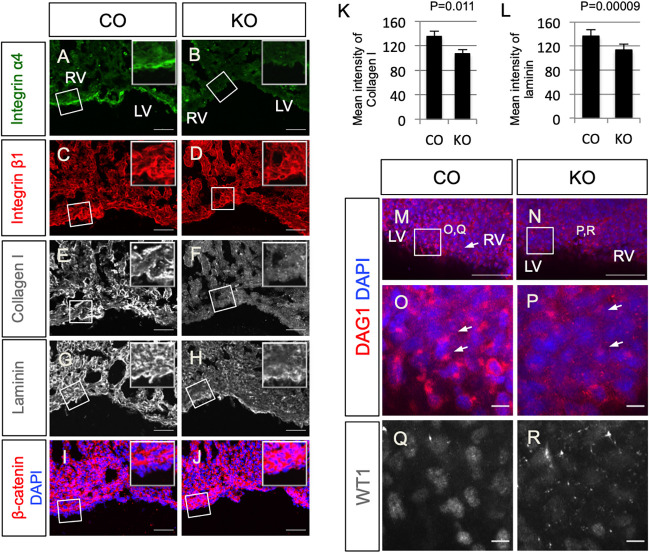
**Deletion of agrin compromises ECM deposition and dystroglycan aggregation in embryonic hearts.** Immunofluorescence staining of integrin α4 (A,B), integrin β1 (C,D), collagen I (E,F), laminin (G,H) and β-catenin (I,J) in serial sections of embryonic hearts of E14.5 littermate control (CO; A,C,E,G,I) and agrin KO (B,D,F,H,J). Note that images in C and E are from the same region of a control section, and images in D and F are from the same region of an agrin KO section. Insets show magnifications of the respective boxed areas. (K,L) Quantification of mean intensity of collagen I (K) and laminin (L) in embryonic heart sections of E14.5 controls and agrin KO. Data represent mean±s.e.m. *n*=3 hearts per group. *P*-values were calculated using an unpaired, two-tailed Student's *t*-test. (M-P) Wholemount immunofluorescence staining of DAG1 to show localization of dystroglycan (DAG1) in the epicardium of littermate control (M,O) and agrin KO (N,P) hearts. (O,P) Magnified views of the boxed areas in M and N. Arrows in M,O,P indicate DAG1 puncta. (Q,R) Immunofluorescence staining of WT1 in the same regions as shown in O and P showing downregulation of WT1 in E16.5 agrin KO (R) compared with the littermate control (Q). LV, left ventricle; RV, right ventricle. Scale bars: 50 μm (A-J,M,N); 10 μm (O-R).

Next, we characterized the expression of other ECM proteins in the agrin KO hearts. We found that epicardial integrin α4 was downregulated in agrin KO hearts (Fig. 5A,B). Collagen I and laminin, both important components of basal membrane ECM, were expressed at lower levels in agrin KO hearts at E14.5, in particular within the myocardium (Fig. 5E-H, Fig. S7A,B). In contrast, expression levels of integrin β1 and PECAM were comparable between controls and agrin KO hearts (Fig. 5C,D, Fig. S7A,B). These results suggest that agrin is important for deposition, assembly or turnover of ECM components in relation to epicardial cell basement membrane connectivity and ongoing EMT. To investigate this further, we examined cytosolic β-catenin expression, which was upregulated in mutant epicardium and myocardium (Fig. 5I,J, Fig. S7C). We measured the average intensity of collagen I and laminin in embryonic heart sections of E14.5 littermate controls and agrin KO and showed both were significantly downregulated in agrin KO (collagen I: 135.23±8.43 in controls versus 106.54±7.38 in agrin KO, *n*=3, *P*=0.011; laminin: 136.57±.2 in controls versus 113.29±9.59 in agrin KO, *n*=3, *P*=0.0009; Fig. 5K,L). Collectively, these data suggest that cells deficient in agrin have tighter connections than those in hearts from littermate controls, and altered ECM constituents associated with compromised EMT potential.

Dystroglycan is a glycoprotein connecting the ECM with intracellular actin (Ervasti and Campbell, 1993) and a known binding partner of agrin (Gee et al., 1994). Therefore, we investigated the distribution of dystroglycan in the embryonic heart using immunofluorescence staining with an antibody against DAG1. At E14.5, the DAG1 antibody detected dense puncta in close proximity to the nuclei of epicardial cells (Fig. 5M,O, arrows). By contrast, in agrin KO hearts, dystroglycan-enriched puncta were either very weak or dispersed (Fig. 5N,P, arrows) and this, in turn, correlated with low WT1 expression indicative of reduced epicardial activation and EMT (compare Fig. 5Q with 5R).

To characterize further the impact of agrin-deficiency on EMT, we investigated epicardial explants derived from E11.5 mouse hearts (Trembley et al., 2016). Epicardium-derived cells arising from agrin KO explants exhibited fewer phalloidin-labeled stress fibers in the periphery and the center of the outgrowth, supporting the hypothesis that agrin is required for epicardial EMT (Fig. S8A-J). Moreover, explants derived from agrin KO hearts exhibited reduced WT1 expression compared with wild-type explants (control 85%±6.2% versus KO 76%±4.5%, *P*=0.03, *n*=4; Fig. S8K). We next investigated the expression pattern of PDPN, which is normally downregulated in cells at the leading edge of epicardial sheets (mesenchymal-like cells), remaining at high levels in the epithelial-like cells closer to the explant (Cao et al., 2017). Although PDPN levels were comparable between KO and control explants, its distribution within the cells differed; PDPN was uniformly localized throughout the cell membrane and cytoplasm in controls, but enriched exclusively in a peri-nuclear pattern in mutant explant cells (Fig. S8L-O, arrows). Smooth muscle myosin heavy chain (SM-MHC) is a protein expressed in vascular smooth muscle cells and is thus used as an indicator for epicardial cell differentiation towards a smooth muscle fate. The number of cells expressing SM-MHC in epicardial cells migrating from explants did not differ between agrin KO and control groups (Fig. S8P-R), indicating that smooth muscle cell differentiation was not affected. Also, in the epicardial cells migrating from explants, there was no difference in the signal or localization of laminin between the agrin KO epicardial cells and the control cells (Fig. S8S,T). In summary, loss of agrin from explants resulted in downregulation of epicardial markers (e.g. WT1, PDPN), defective ECM organization and compromised epicardial cell migration, but did not affect their differentiation potential towards smooth muscle.

### Agrin promotes EMT in cultured human and mouse epicardial cells

**Fig. 6.**
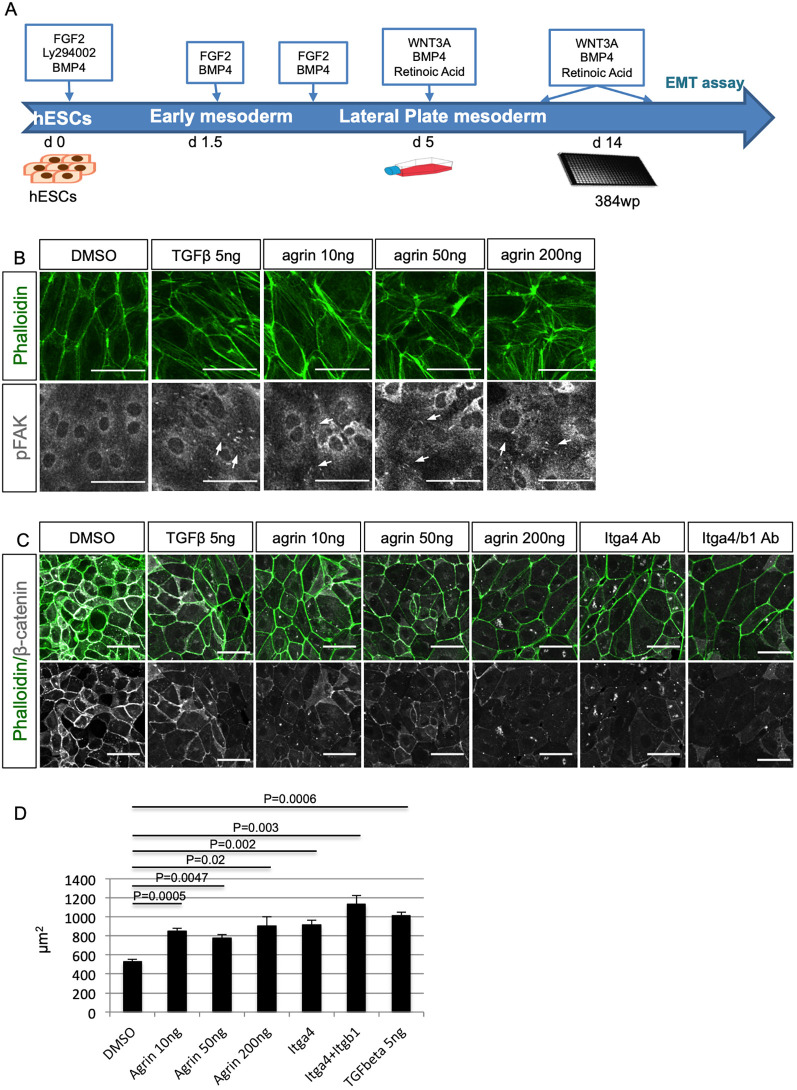
**Agrin promotes EMT by enhancing pFAK and decreasing β-catenin.** (A) Schematic showing the differentiation protocol of human epicardial cells (hEPDCs) from hESCs. d, day. (B) Treatment of human epicardial-like cells with TGFβ and agrin resulted in enhancement of stress fibers (phalloidin, green) and pFAK (white). (C) Immunofluorescence staining for stress fibers (phalloidin, green) and β-catenin (white) on human epicardial-like cells treated with agrin or blocking antibody against integrin α4 and integrin β1. (D) Addition of agrin enhanced cell size, indicating EMT. Data represent mean±s.e.m. *n*=3 treatments per group. *P*-value was calculated using one-way ANOVA among all the groups (the *P* value at the top). Two-tailed Student's *t*-tests were used to examine differences between groups (all the other *P* values). Scale bars: 50 μm.

Our data revealed that loss of agrin impacts on mouse epicardial development, coronary vasculature formation and epicardial EMT. To characterize further the role of agrin in EMT and assess a potential conservation of this function in the human epicardium, we adopted an *in vitro* differentiation protocol generating epicardial-like cells from human embryonic stem cells (hESCs) (Iyer et al., 2016; Fig. 6A). Following treatment with 5 ng/ml TGFβ, a known inducer of EMT, for 72 h, the human epicardial-like cells presented clear morphological changes, such as enhanced stress fiber density. Importantly, we observed a similar phenotype after treating the cells with increasing concentrations of recombinant agrin (10 ng/ml, 50 ng/ml, 200 ng/ml) (Fig. 6B, upper panels). In addition to stress fiber density, we assessed the levels of phosphorylation of FAK at tyrosine 397 (pFAK Y397), which connects the ECM signals and intracellular signaling through integrins and is a key regulator of cell migration, an important process in cells undergoing EMT (reviewed by Mitra et al., 2005; Larsen et al., 2006). Treatment with 5 ng/ml TGFβ enhanced the localization of pFAK Y397 at focal adhesions (Fig. 6B, lower panels). Similarly, agrin also induced focal adhesion localization of pFAK Y397, suggesting agrin had a similar effect as TGFβ in promoting human epicardium-like cells towards a mesenchymal fate (Fig. 6B).

In the human *in vitro* model, the epithelial-like cells exhibited high numbers of β-catenin-positive cell-cell junctions. Treatment with 5 ng/ml TGFβ downregulated the localization of β-catenin at the cell-cell junctions (Fig. 6C). An equivalent downregulation was also observed in human epicardium-like cells following exogenous agrin treatment (10 ng/ml, 50 ng/ml, 200 ng/ml) consistent with an EMT-promoting effect. The effect of agrin was dose dependent and more potent than TGFβ at high dosage. Incubation of human epicardial-like cells with the blocking antibody natalizumab (‘Itga4 ab’), which specifically targeted epicardial α4 integrin in this instance, also reduced β-catenin localization at cell-cell junctions. Treatment with combined blocking antibodies against the epicardial-specific α4β1 integrin had a stronger effect in decreasing β-catenin at cell-cell junctions (Fig. 6C). Addition of agrin or integrin-blocking antibodies also significantly enhanced the cell size, phenocopying TGFβ treatment (Fig. 6D). In combination, the effect of agrin on enhancing stress fibers and pFAK at focal adhesions, as well as downregulation of β-catenin at the cell-cell junction indicates that agrin promotes EMT and an evident mesenchymal cell fate.

To confirm the *in vitro* effects of agrin on EMT and cell migration, we used the immortalized mouse epicardial cell line MEC1 (Li et al., 2011) in a wound-healing assay. Addition of agrin, following an automated scratch assay, at 200 ng/ml promoted wound healing at an equivalent rate to 5 ng/ml TGFβ, with 75% of the scratch closed after 64 h (Fig. S9). Blocking antibody combinations against integrin α4β1 also promoted wound healing to the same level as agrin treatment. In contrast, treatment with an agrin-blocking antibody decreased wound healing to 42%, compared with 55% following control PBS treatment. Interestingly, blocking antibody against the agrin binding partner dystroglycan severely reduced the mouse EPDC migration to 20%, and this effect could not be rescued by addition of TGFβ (Fig. S9). These data suggest that agrin has comparable, but independent, effects to TGFβ in promoting EMT and migration of mouse epicardial cells resulting in enhanced wound closure.

### Blocking agrin decreases YAP signaling in EPDCs

To investigate additional pathways that agrin treatment might impact upon in the context of epicardial EMT, we examined Hippo-YAP signaling, previously shown to be essential for the development of the epicardium and epicardial cell differentiation (Xiao et al., 2018) and to act as a potent inducer of EMT in other cell types, most notably cancer (Cheng et al., 2020). More specifically, YAP activation has been shown to drive EMT-like processes during cardiac regeneration (Aharonov et al., 2020) and agrin treatment has been shown to enhance YAP nuclear localization in cardiomyocytes within the infarcted heart (Bassat et al., 2017). Moreover, agrin knockdown in liver cancer cells resulted in phosphorylation of YAP (pYAP) at Ser127 and repression of downstream genes (Chakraborty et al., 2017). We examined YAP subcellular localization and phosphorylation in human epicardial-like cells treated with agrin and agrin-blocking antibody. Immunostaining revealed that agrin treatment moderately enhanced YAP in both nuclear and cytosol compartments, whereas TGFβ showed minimal effect (compare Fig. S10A, S10B and S10C). Treatment with agrin-blocking antibody, by contrast, significantly decreased YAP nuclear localization and enhanced cytosol localization to bring about YAP inactivation (Fig. S10D). Both agrin and agrin-blocking antibody enhanced YAP phosphorylation at Ser127 (pYAP) as the precursor for cytoplasmic translocation, with antibody treatment revealing a significantly stronger effect (Fig. S10E). These data suggest that blocking agrin significantly increases the pYAP/YAP ratio, suppressing Hippo-YAP signaling in epicardial cells to potentially contribute to impaired EMT.

### Agrin regulates EMT through aggregation of dystroglycan to the Golgi apparatus maintaining basement membrane and cytoskeletal connectivity

**Fig. 7.**
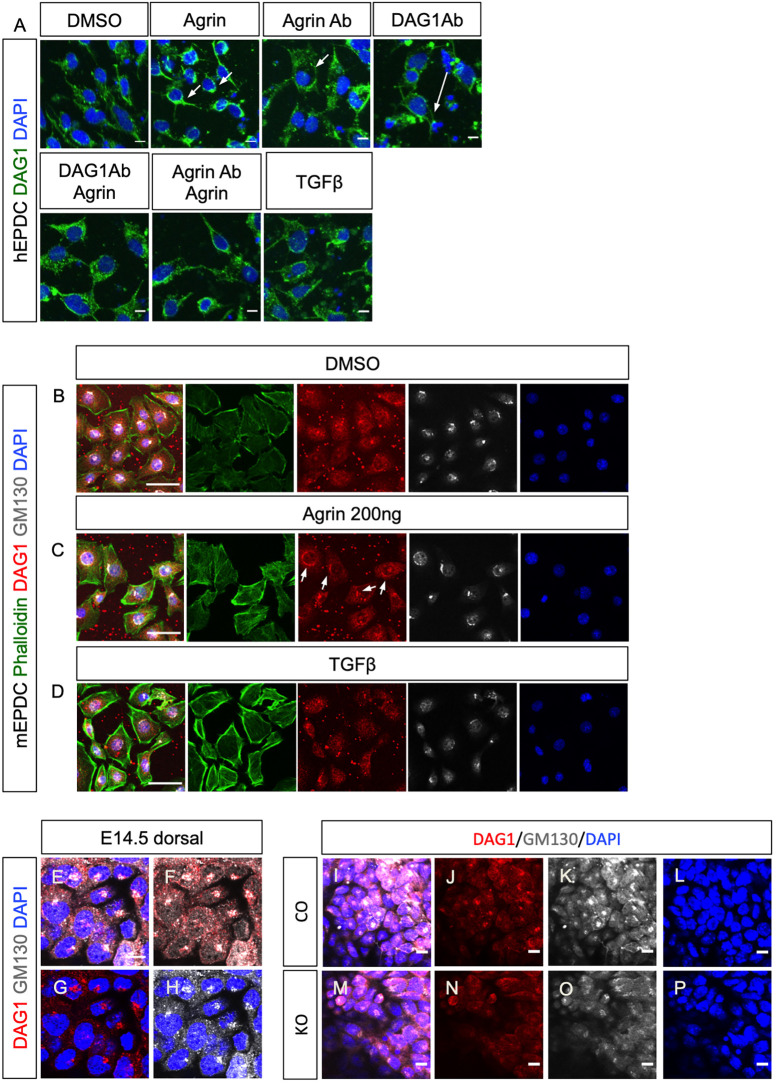
**Agrin aggregates DAG1 to the Golgi apparatus in epicardial cells.** (A) Immunofluorescence staining for DAG1 (green) and DAPI (blue) of human epicardial-like cells treated with agrin or blocking antibodies. Compared with the negative DMSO control, DAG1 was detected in proximity of nuclei when 200 ng/ml agrin (Agrin) was added. Blocking agrin with agrin blocking antibody (Agrin Ab) resulted in dispersal of dystroglycan (arrow). Treatment with blocking antibody against DAG1 (DAG1Ab) also resulted in dystroglycan dispersal (arrow). These effects were partially rescued by addition of agrin (DAG1Ab Agrin and Agrin Ab Agrin). TGFβ treatment did not aggregate dystroglycan to proximal nuclei. (B-D) Immunofluorescence staining with DAG1 antibody (red) and GM130 antibody (white) showing that agrin aggregates dystroglyan to the Golgi apparatus in epicardial cells. Addition of agrin caused aggregation of dystroglycan (arrows in C), which overlapped with the Golgi apparatus labeled with GM130 (white). TGFβ did not aggregate dystroglycan (D). (E-H) Wholemount immunofluorescence staining in E14.5 hearts showing that dystroglycan in epicardial cells aggregated in Golgi apparatus labeled with GM130. (I-P) Wholemount immunofluorescence staining with DAG1 and GM130 antibody of E14.5 control (I-L) and agrin KO (M-P) hearts showing disorganized Golgi in the epicardial cells of agrin KO. LV, left ventricle; RV, right ventricle. Scale bars: 10 μm.

To assess further a role for dystroglycan in agrin-mediated EMT, we investigated the distribution of dystroglycan in human epicardium-like cells following exogenous agrin treatment (Fig. 7A). In cells treated with DMSO, dystroglycan was detected at low levels around nuclei, whereas stimulation with 200 ng/ml agrin led to aggregation of dystroglycan in dense perinuclear puncta (Fig. 7A, Agrin, arrows). In contrast, blocking endogenous agrin with a neutralizing antibody resulted in the dissociation of dystroglycan puncta (Fig. 7A, Agrin Ab, arrow). Blocking dystroglycan with a DAG1 antibody had a similar effect to agrin antibody treatment (Fig. 7A, DAG1Ab, arrow), suggesting that agrin signals through dystroglycan in this epicardial cell model. Combined treatment of cells with agrin together with a DAG1 antibody or agrin antibody partially rescued the dissociation of dystroglycan puncta from the nuclei (Fig. 7A, DAG1Ab Agrin, Agrin Ab Agrin). In contrast with agrin, TGFβ did not aggregate dystroglycan around the nuclei despite promoting EMT of the human epicardial-like cells (Fig. 7A, TGFβ).

The subcellular pattern of aggregation induced by agrin suggests that the dystroglycan may localize to specific organelles within the cell. A previous study implicated Golgi-resident glycosyltransferases fukutin and fukutin-related protein as being required for post-translational modification of dystroglycan (Esapa et al., 2002), suggesting that dystroglycan may localize in the Golgi apparatus. To test this hypothesis, we examined dystroglycan expression in conjunction with the Golgi marker GM130 (also known as GOLGA2) (Nakamura et al., 1995, 1997) following treatment with exogenous agrin. Under basal/control conditions, dystroglycan was detected on the plasma membrane, with mild enrichment in the nuclear envelope and the Golgi apparatus labeled with GM130 (Fig. 7B). Treatment with 200 ng/ml agrin led to enhanced stress fibers (compare Fig. 7B and 7C) and condensed aggregation of dystroglycan within the GM130-positive Golgi apparatus (Fig. 7C). TGFβ treatment also enhanced the incidence of stress fibers (compare Fig. 7D and 7B) but did not induce aggregation of dystroglycan within the Golgi apparatus (Fig. 7D).

We further examined the morphology of the Golgi apparatus within epicardial cells *in vivo*. In E14.5 hearts, GM130 labeled the Golgi apparatus as dense puncta within the developing epicardium. Dystroglyan, detected with DAG1 antibody, overlapped with GM130^+^ puncta, indicating that dystroglycan is also aggregated in the Golgi apparatus *in vivo* (Fig. 7E-H). We then examined the Golgi apparatus of epicardial cells in E14.5 agrin KO hearts and found they were poorly defined and lacked discrete structure, although dystroglycan remained colocalized with GM130 (Fig. 7I-P; see also Fig. 5N,P, arrows, for dispersed dystroglycan puncta in the agrin KO epicardium). This suggested that loss of agrin compromises the morphology of the Golgi apparatus in epicardial cells *in vivo*. Collectively, these data identify dystroglycan as downstream of agrin signaling in promoting epicardial EMT and suggest that dystroglycan enrichment in the Golgi apparatus may act to maintain ECM basement membrane-cytoskeletal integrity within the epicardium.

## DISCUSSION

In this study, we have characterized the role of ECM components during heart development and specifically in the essential process of epicardial EMT. Regions of active EMT were associated with the deposition of specific matrix components in the epicardial layer. Specifically, we have identified a novel and essential role for the basement membrane proteoglycan agrin during epicardial development. Our data reveal that agrin promotes epicardial EMT in both mouse and human models, through aggregation of dystroglycans to the Golgi apparatus, connecting extracellular signals from ECM with intracellular pathways, such as Hippo-YAP signaling. The function of ECM in development has been studied previously, but there has been limited insight into ECM signaling and the regulation of cellular processes in the forming heart.

### Epicardial EMT and the ECM

Using SEM, we observed morphological heterogeneity within epicardial cells across different development stages. In the E13.5 and E14.5 epicardium, we observed large flat cells tightly connected with their neighbors in addition to much smaller, round cells that were clearly separated from each other. The morphologies of these cells are consistent with the definition of epithelial cells and mesenchymal cells (Yang et al., 2020), suggesting we observed *de novo* EMT at high resolution over the outer apical surface of the epicardial layer. Recently, Velecela et al. (2019) described epicardial cells with similar morphologies under SEM as indicative of relative maturity; however, our data provides novel insight into the co-existence of epicardial cells of different morphologies, correlating with EMT status rather than maturity.

Consistent with the observations from our SEM analyses, active and inactive regions of EMT were associated with the presence of key ECM components. We found that integrin α4 was prominently downregulated in regions of high EMT. This observation is supported by a previous report that knock down of integrin α4 in chicken epicardial cells leads to a highly invasive phenotype and increased migration of cells into the underlying myocardium of chicken-quail chimera (Dettman et al., 2003). However, loss of agrin in the current study resulted in downregulation of integrin α4 and decreased EMT, indicating that integrin α4 has additional roles beyond restraining epicardial cells. It is plausible that integrin α4 acts as an anchoring molecule that secures epicardial cells to the basement membrane and a link with the extracellular environment to relay signals for proliferation and maintenance. Laminin is one of the main components of the basement membrane (reviewed by Yurchenco, 2015) and we observed that in inactive EMT regions epicardial laminin was continuous between the epicardial cells and the myocardium, suggesting an integral basement membrane between these two layers of the developing heart wall. In contrast, in regions of active EMT laminin was reduced and discontinuous among epicardial cell clusters. The difference in the deposition of laminin and α4 integrin in regions of low- versus high-EMT suggests that the basement membrane and interstitial ECM are highly dynamic developmental structures.

### Agrin is a novel regulator of epicardium development and EMT

The impact of agrin loss on the migration of WT1^+^ cells indicates agrin is an essential regulator of epicardial development. Our SEM analysis of agrin KO epicardial cells revealed several morphological changes compared with wild-type littermates, including fewer protrusions at cell-cell junctions and on the cell surface. These protrusions have not been described before within the forming epicardium and their function remains unknown as applied to other cell types. Based on our observation that cells undergoing EMT have more protrusions compared with cells with an epithelial morphology, and that epicardial cells of agrin KO hearts have fewer protrusions than controls, we hypothesize that these protrusions are correlated with EMT activity, possibly as a result of cytoskeleton movement. Loss of agrin compromised several ECM components of the epicardium, such as integrin α4, podoplanin and, most importantly, WT1, which is a master transcriptional regulator of epicardial EMT (Vieira et al., 2017). Laminin and collagen I were also downregulated in agrin KO mutants, not only in the epicardium but also in myocardium, indicating a more global role for agrin in ECM organization. The upregulation of cytosolic β-catenin in the epicardium, and cells adjacent to the epicardium, suggests decreased EMT potential in these cells. Interestingly, a previous report revealed that agrin contributes to the blood-brain barrier by stabilizing the localization of tight junction proteins, such as VE-cadherin (cadherin 5), β-catenin and ZO1 at cell-cell junctions (Steiner et al., 2014). In contrast, in C2C12 myoblasts overexpression of β-catenin blocked the effect of agrin on the aggregation of the acetylcholine receptor (Wang et al., 2008), suggesting an antagonistic relationship between agrin and β-catenin.

The coronary vasculature defects of the agrin KO mutant phenocopy those observed in WT1 knockout hearts (von Gise et al., 2011), and are potentially a consequence of compromised epicardial EMT. However, agrin is also an important component of the basement membrane of the vasculature. The abnormal arrangement of PECAM^+^ endothelial cells of the coronary vessel in agrin KO hearts is more likely a direct consequence of agrin absence in blood vessel endothelium. Indeed, agrin can stabilize vascular endothelial growth factor receptor 2 (VEGFR2; also known as KDR) in the ECM and thus promote angiogenesis (Njah et al., 2019).

### Agrin promotes EMT through aggregation of dystroglycan

Agrin has multiple isoforms, including membrane-anchored and secreted variants (Burgess et al., 2000; Neumann et al., 2001). Agrin regulates intracellular signaling pathways through binding to the low-density lipoprotein receptor 4 (LRP4) and muscle-specific tyrosine kinase (MuSK), which has been characterized in detail in muscle-neuron junctions (Zhang et al., 2008). Dystroglycan is another known binding partner of agrin (Gee et al., 1994; Scotton et al., 2006) and is an essential component of the dystrophin-associated glycoprotein complex (DGC). Both α- and β-dystroglycans are encoded by the *DAG1* gene (Ibraghimov-Beskrovnaya et al., 1992). Dystroglycan links the ECM with the actin cytoskeleton (Ervasti and Campbell, 1993). In cardiomyocytes, dystroglycans associate with phosphorylated YAP, the Hippo pathway effector, and inhibit cardiomyocyte proliferation (Morikawa et al., 2017). Agrin promotes cardiomyocyte proliferation in post-myocardial infarction hearts through DAG1 and YAP (Bassat et al., 2017) and in our study we observed a requirement for agrin to ensure YAP activation in the context of epicardial EMT. Although dystroglycans are usually detected in the cell membrane, a nuclear localization signaling (NLS) sequence has been identified in β-dystroglycan (Lara-Chacón et al., 2010). There is also report that dystroglycans can be translocated to the nuclear membrane in C2C12 myoblast cell lines, playing an important role in maintaining the integrity of the nuclear envelope (Gracida-Jiménez et al., 2017; Vélez-Aguilera et al., 2018). Our results suggest that agrin regulates epicardial EMT through aggregation of dystroglycan. Disaggregation and dispersal of dystroglycan was observed in agrin KO hearts coincident with impaired EMT, suggesting that the agrin-dystroglycan signaling axis is important for maintaining ECM/basement membrane and epicardial cell cytoskeletal integrity and intracellular signaling (e.g. Hippo-YAP) cross-talk to ensure mesenchymal transition. In cultured epicardial cells as well as embryonic hearts, this aggregation appears to be via the Golgi apparatus. A previous study reported that Golgi compaction is related to the mesenchymal status of lung adenocarcinoma cell lines (Tan et al., 2017), implying that the dynamics of the Golgi apparatus may be an important factor influencing EMT. Dystroglycans must be glycosylated in the Golgi apparatus to acquire function (Michele et al., 2002), thus another probability is that agrin regulates downstream signals through post-translational modification of dystroglycans. To date, the role of dystroglycan in heart development and epicardium formation has not been characterized. A glycosylation-deficient dystroglycan and cardiac myocyte-specific deletion of *DAG1* have previously been shown to lead to interstitial fibrosis and cardiomyopathy (Michele et al., 2009). A point mutation, T192-M, in the *DAG1* gene has also been identified in a human patient with limb-girdle muscular dystrophy and a mouse model carrying this mutation recapitulates the abnormalities in human. This mutation impairs the receptor function of dystroglycan by inhibiting its post-translational modification (Hara et al., 2011). It will be of interest to investigate how heart development may be affected, either following conditional loss of function of *DAG1* or the introduction of an analogous *DAG1* point mutation in the developing epicardium.

In summary, our results identify agrin and its receptor dystroglycan as important regulators of EMT in the embryonic epicardium and provide novel insights into the essential function of the ECM in the developing mammalian heart.

## MATERIALS AND METHODS

### Mouse lines

Genetically modified mouse lines were kept in a pure C57BL/6 background. *Agrin^fl/fl^* mice were crossed with *PGK-Cre* mice to generate *Agrin^+/−^* progenies. *Agrin^−/−^* (Agrin KO) mice were generated by intercrossing *Agrin^+/−^* males and females. *Wt1-CreERT2* mice were crossed with *Wt1-GFPCre* to generate *Wt1^KO^* embryos. For timed-mating experiments, mice of breeding ages were paired in breeding cages and vaginal plugs were checked in the morning until the females were plugged. The date of a vaginal plug was set as E0.5. Mice were housed and maintained in a controlled environment by the University of Oxford Biomedical Services. All animal experiments were carried out according to the UK Home Office project licence (PPL) 30/3155 and PPDE89C84 compliant with the UK animals (Scientific Procedures) Act 1986 and approved by the local Biological Services Ethical Review Process. See Table S1 for details of strains used.

### Scanning electron microscopy

Samples were fixed in 2.5% glutaraldehyde, pH 7.4, at 37°C after dissection then stored at 4°C overnight. After rinsing with PBS, the samples were post-fixed with 1% OsO_4_ for 1 h then rinsed with distilled water. After dehydration through an ethanol gradient, the samples were dried with a critical point dryer. They were then mounted on a stub with carbon tape and coated with gold-sputter for imaging with a Zeiss Sigma 300 FEG-SEM operating at 2 kV.

### Fluorescence immunohistochemistry and quantification

Embryos were harvested at the required stages and their hearts were dissected in PBS and fixed with 4% paraformaldehyde (PFA; Santa Cruz Biotechnology, 281692) overnight. For wholemount staining, samples were washed in 0.3% Triton X-100 in PBS and blocked in 1% bovine serum albumin (BSA; Merck, 05470), 0.3% TritonX-100 in PBS for at least 2 h. The samples were then incubated with primary antibodies (see Table S2) in the blocking solution overnight at 4°C. On the second day, the samples were washed at least five times in 0.3% Triton X-100 in PBS. The samples were then incubated with secondary antibodies (see Table S2) and DAPI (Invitrogen, 62248) diluted in PBS overnight at 4°C. The samples were then washed with PBS at least five times the next day and mounted in 50% glycerol in PBS for scanning with an Olympus FV1000 confocal microscope.

To prepare tissue sections, the fixed embryonic hearts were embedded in OCT compound (Agar Scientific, AGR1180) embedding medium. Serial sections were prepared with a cryostat microtome. Cells or heart explants were cultured in 10% fetal bovine serum DMEM (Life Technologies) on coverslips coated with 0.1% gelatin. For sections and cell cultures, glass slides or coverslips were washed twice with PBS to remove the OCT then permeabilized with 0.5% Triton X-100 in PBS for 10 min and washed with PBS afterwards. The sections or cells were then blocked in 10% donkey or goat serum, 1% BSA and 0.1% Triton X-100 in PBS for at least 1 h at room temperature. Next, samples were incubated with primary antibodies (see Table S2) diluted in the blocking solution at the concentration indicated above overnight at 4°C. The next day, samples were washed three times in 0.1% Triton X-100 in PBS for 10 min then incubated with corresponding fluorescent secondary antibodies diluted at 1:200 in 0.1% Triton X-100 in PBS for 1 h. After three washes with 0.1%Triton X-100 and 10 min of DAPI staining, slides were mounted in 50% glycerol in PBS and imaged with Olympus FV1000 confocal microscope. Images were analyzed with Fiji (NIH) and AngioTool (Zudaire et al., 2011).

To quantify WT1 cells in the epicardium, WT1^+^ nuclei and all DAPI-stained nuclei labeled with PDPN were manually counted. For each sample, two to four regions from each section were randomly selected for quantification and two or three sections were analyzed per heart (*n*=3 hearts per group). To quantify WT1^+^ cells in myocardium area, WT1^+^ nuclei in a defined region were manually counted using ImageJ. Total DAPI area was measured using ImageJ. The ratio of WT1^+^ nuclei number against total DAPI area (10^4^ μm^2^) was calculated. Two or three regions were selected from each section, and two to four sections were analyzed for each sample. Data represent mean±s.e.m. (*n*=3 hearts per group). Significant differences were calculated using an unpaired, two-tailed Student's *t*-test between control and KO group. To quantify WT1^+^ cells in E16.5 epicardium, WT1^+^ nuclei and total DAPI nuclei in defined regions were manually counted in ImageJ. Two regions from similar locations were selected from each sample (*n*=3 hearts were analyzed per group); data represent mean±s.e.m. and the *P*-value was calculated using an unpaired, two-tailed Student's *t*-test.

For the immunofluorescence intensity quantification shown in Fig. 5, the signal intensity of the antibody staining and of DAPI staining was measured with ImageJ. Staining of the same set of markers was performed and scanned at the same time to avoid variations. Two to four serial sections were analyzed for each sample (*n*=3 hearts were analyzed per group); data represent mean±s.e.m. and the *P*-value was calculated using an unpaired, two-tailed Student's *t*-test.

To quantify cell area as shown in Fig. 6, total cell numbers (nuclei numbers) were counted in a defined region and average cell size was calculated. Four or five regions were counted for each treatment (*n*=3 treatments per group). Data represent mean±s.e.m. and the *P*-value was calculated using a one-way ANOVA for comparisons across all the groups. For comparisons between two groups, a two-tailed Student's *t*-test was used.

### Immunoblotting

Human and mouse EPDCs were lysed in RIPA buffer. Western blotting was performed using standard SDS-PAGE methods (Bio-Rad). YAP (Novus Biologicals, NB110-58358, 1:1000), pYAP (Cell Signaling Technology, 4911, 1:1000) and β-actin (Abcam, Ab8227, 1:2000) antibodies were used to detect corresponding proteins. Bands were visualized using HRP-conjugated secondary antibodies (anti-mouse IgG: Sigma-Aldrich, NAP31V; anti-rabbit IgG: Sigma-Aldrich, NA9340) and enhanced chemiluminescence detection (ThermoFisher).

### hESC-derived epicardium-like cell differentiation

hESCs (H9 line) were cultured in mTeSR1 basal medium (Stem Cell Technologies), in 6-well plates coated with Matrigel (Corning Matrigel Matrix hESC-qualified). Once hESCs reached 75% confluency, the differentiation was initiated, based on the method by Iyer et al. (2016), with certain modifications. Specifically, for the CDM-PVA (chemically defined medium-polyvinyl alcohol) basal cardiac differentiation medium, human insulin was purchased from Sigma-Aldrich and was used at a final concentration of 7 μg/ml, and WNT3A (R&D Systems) was used at a concentration of 50 ng/ml. Furthermore, on day 5 of differentiation, when the lateral plate mesoderm cells were passaged to generate epicardium, the cells were seeded at a 75×10^4^/cm^2^ density with additional supplementation of 10 μM Rock inhibitor (Y-27632, Abcam). The next day, Rock inhibitor was removed by replenishing with cardiac differentiation medium, CDM-PVA supplemented with WNT3A (50 ng/ml), BMP4 (50 ng/ml, R&D Systems) and all-trans retinoic acid (4 μM, Sigma-Aldrich). The differentiation continued until day 14, with the media being replenished once more at day 11, and topped up with fresh solution as required when the color of the cell media turned orange.

### Mouse EPDC EMT induction

Embryonic immortalized mouse epicardium-derived cells (EPDCs; PromoCell, C-12216) were cultured in growth medium comprising Dulbecco's Modified Eagle Medium (DMEM), high glucose, GlutaMAX (Gibco, 10566016) supplemented with 0.1% Pen Strep (Gibco, 15140-122), 0.1% Insulin-Transferrin-Selenium (ITS; Gibco, 41400045), 10 U/ml Interferon (IFN) γ (Peprotech, 315-05) and 10% heat-inactivated fetal bovine serum (HI-FBS; Gibco, 10500). To induce EMT, cells were seeded at a density of 1.4×103 cells/cm^2^ and treated with 2.0 µg/ml human recombinant TGFβ (R&D Systems; 240-B) or other indicated reagents 16 h later for a duration of 30 h. Cells were then fixed in 4% PFA prior to immunofluorescence assessment.

### Wound healing assay

To assess wound closure capacity, 10,000 mEPDC were seeded per well of a 96-well plate 16 h prior to scratch initiation, by which point cells have formed a confluent monolayer. A uniform scratch was applied to all wells using the 96-pin WoundMaker (Essen BioScience), and wells were washed twice with media to remove floating cells. Immediately following wounding, cell treatments (12 wells per group) were added to the growth medium and the plate incubated at 37°C, 5% CO_2_ until complete wound closure was achieved. Wells were imaged every 4 h using the live content imaging system IncuCyte HD (Essen BioScience) and the integrated analysis algorithm automatically masks each image to identify cell-free and cell-occupied areas in order to calculate relative wound density (RWD; the ratio of the occupied area to the total area of the initial scratched region) for each time point.

### Statistical analysis

All data are presented as mean±s.e.m. Statistical analysis was performed using GraphPad Prism 8 software. The statistical significance between two groups was determined using unpaired two-tailed Student's *t*-tests; these included an *F*-test to confirm the two groups had equal variances. Among three or more groups, one-way analysis of variance (ANOVA) followed by Tukey's multiple comparison test was used for comparisons. A value of *P*≤0.05 was considered statistically significant.

## Supplementary Material

Supplementary information

Reviewer comments
