# Supplementary material for: The extracellular matrix protein agrin is essential for epicardial epithelial-to-mesenchymal transition during heart development
Source: Development. 2021 May 10;148(9):dev197525. doi: 10.1242/dev.197525 (PMC8172119; doi:10.1242/dev.197525)
Supplement: Supplementary information [file develop-148-197525-s1.pdf]

## Figure S1

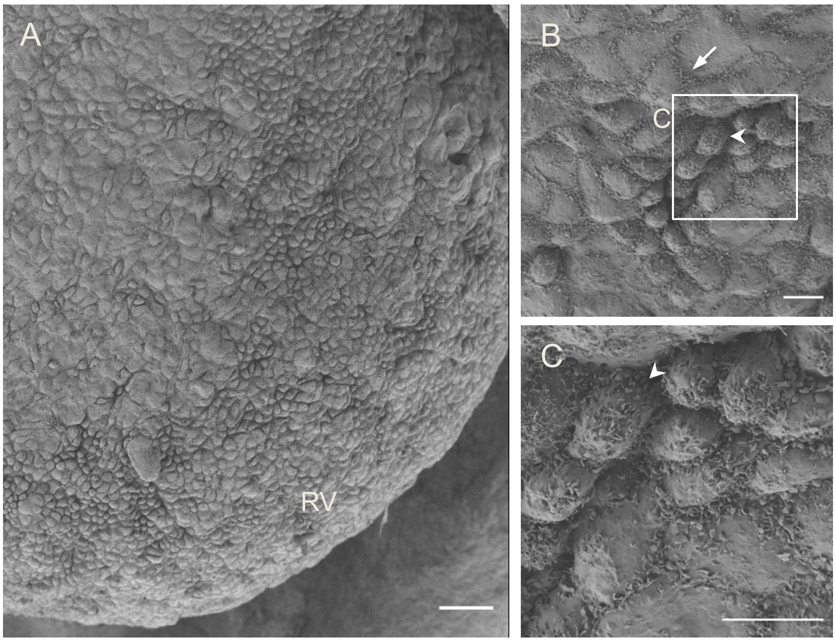


**Figure S1. Cells with epithelial morphology and mesenchymal morphology within the embryonic epicardium.** (A): the dorsal surface of an E13.5 embryonic heart, showing epicardial cells with different morphologies. (B): Magnified epicardium focusing on large and flat, epithelial-like cells (white arrow) and small, pillar-shaped, dispersed mesenchymal-like cells (white arrowhead). (C): magnified view of the inset box from (B). Scale bars: A, 50 μm. B, C: 10 μm. RV: right ventricle.

# Figure S2

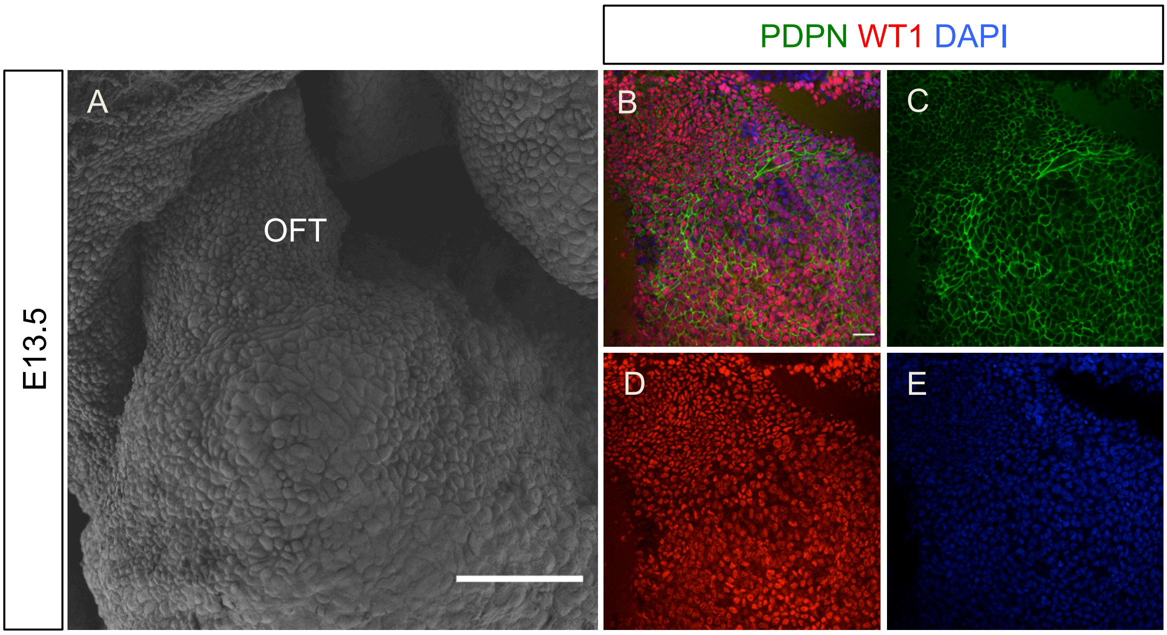


**Figure S2. Morphological differences between epicardial cells in the OFT.** (A): the OFT of an E13.5 heart under SEM: note the presence of distinct small, round cells and large, flat cells. (B-E), Whole-mount immunofluorescent staining of E13.5 for podoplanin (PDPN; green) and WT1 (red). Scale bars: 50 μm. OFT: outflow tract.

# Figure S3

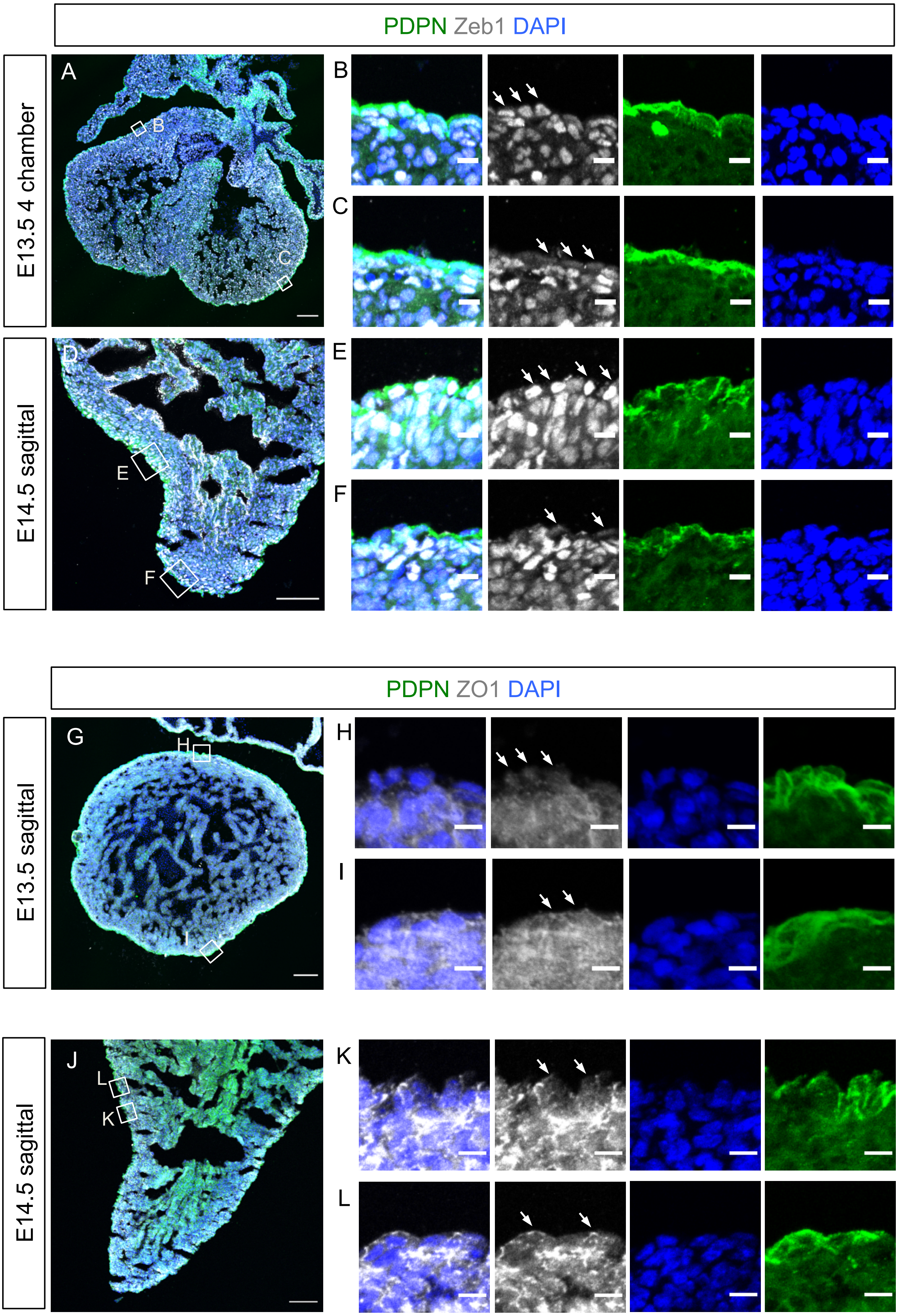


Development • Supplementary information

**Figure S3. Heterogenous EMT status of epicardial cells.** (A-F): immunofluorescent staining of ZEB1 (white), Podoplanin (PDPN; green) and DAPI (blue) of a section from a E13.5 heart (A) and a section from E14.5 heart (D). (B and C) are magnified view from the inset boxes in (A). (E and F) are magnified view from the inset boxes in (D) Epicardial cells express high level of Zeb1 (B, E white arrows) and low level of Zeb1 (C, F, white arrows). (G-L): immunofluorescent staining of ZO1 (white), Podoplanin (green) and DAPI (blue) of a section from a E13.5 heart (G) and a section from a E14.5 heart (J). (H and I) are magnified view from the inset boxes in (A). (K and L) are magnified view from the inset boxes in (J). In (H and K), epicardial cells bear low and discontinuous ZO1 expression (white arrows). In (I and L), ZO1 is detected in cell junctions and apical side (white arrows). Scale bars: A, D, G, J: 50 μm. B, C, E, F, H, I, K, L: 10 μm.

# Figure S4

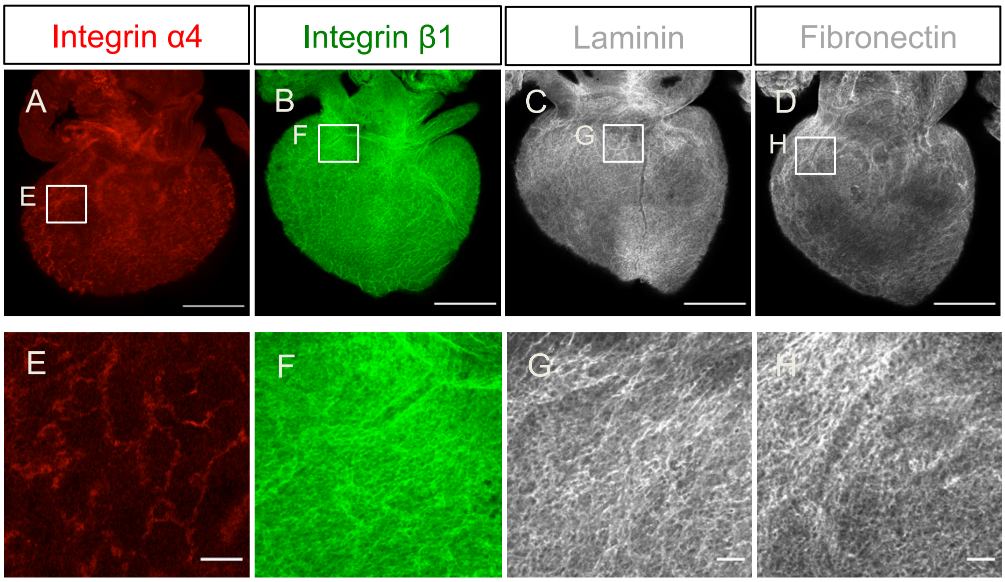


**Figure S4. Expression of extracellular matrix components within the embryonic epicardium.** (A-D). Wholemount immunofluorescent staining of integrin α4 (red), integrin β1 (green), laminin (white) and fibronectin (white) on E14.5 embryonic hearts. E-H: magnified view of the inset boxes from A-D. Scale bars: A-D, 500 μm. E-H: 50 μm.

# Figure S5

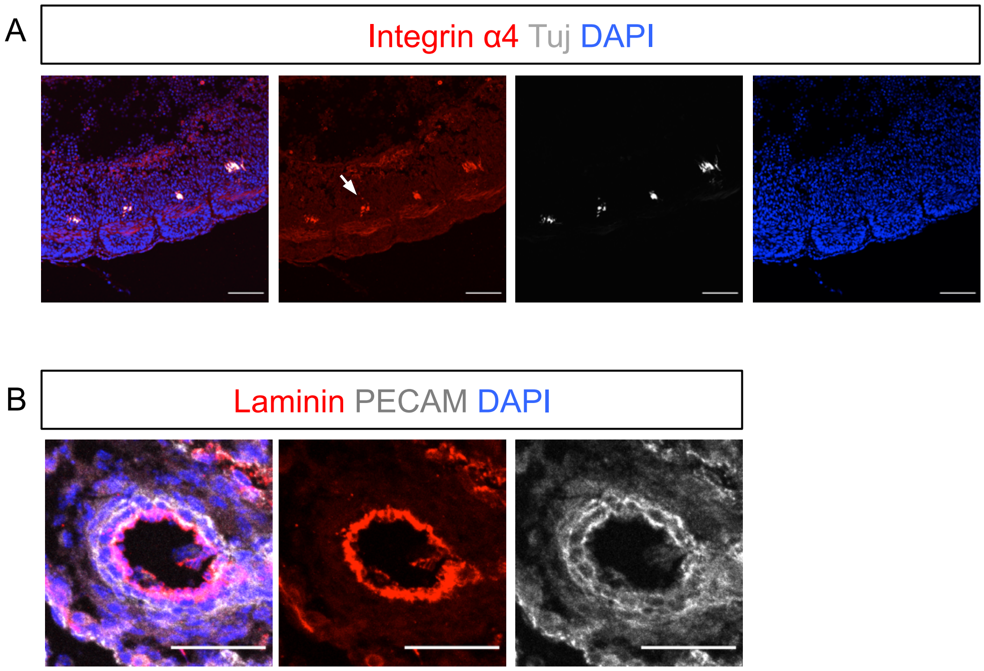


**Figure S5. Validation of integrin α4 and laminin antibodies.** (A): Immunofluorescent staining of Integrin α4 (red), Neuronal β-tubulin (TuJ1) and DAPI (blue) of a section from a E10.5 embryo. Integrin α4 overlaps with TuJ in somites. (B): Immunofluorescent staining of laminin (red) and PECAM (white) of a blood vessel. Scale bars: 50 μm.

Development • Supplementary information

## Figure S6

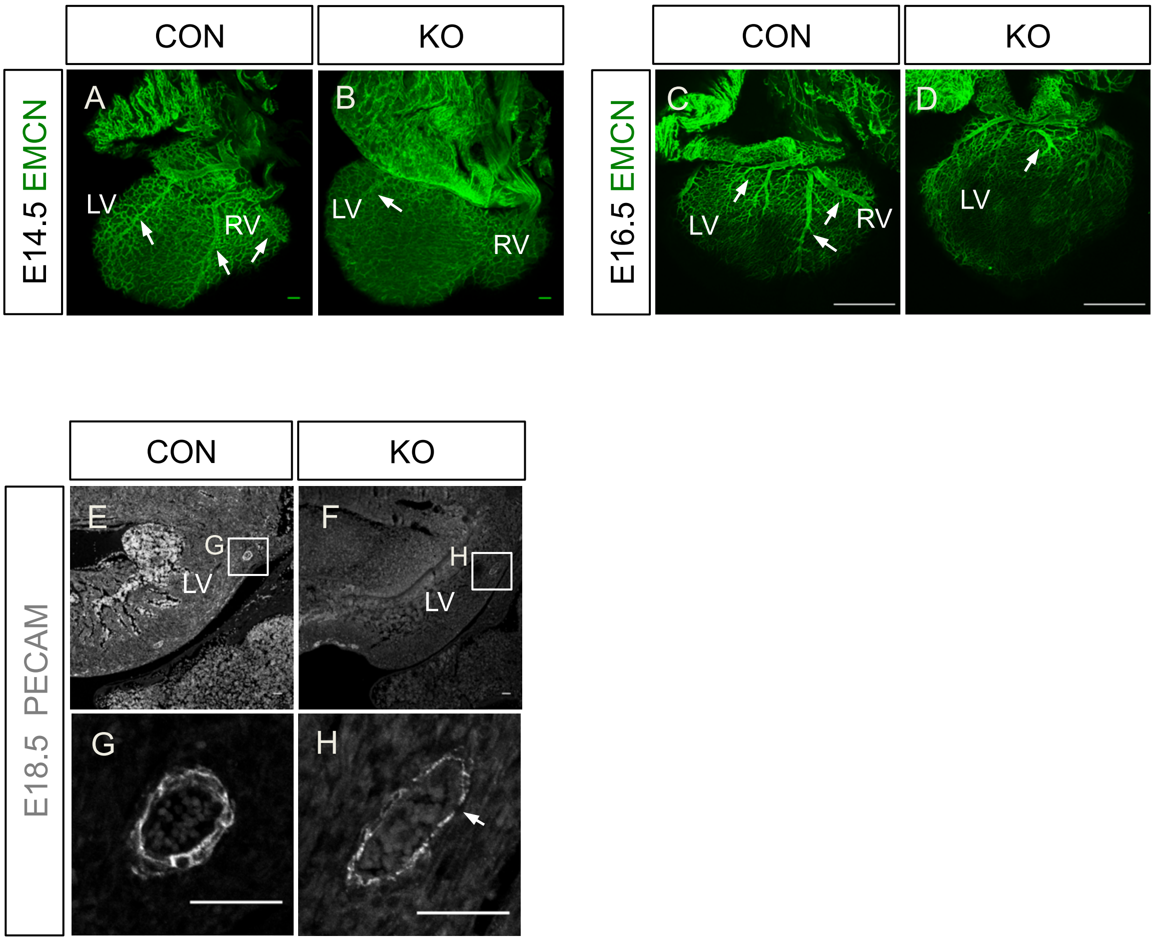


**Figure S6. Coronary vasculature defects in agrin KO hearts.** (A-D), whole-mount immunofluorescent staining for EMCN (green) of E14.5 (A, B) and E16.5 (C, D) littermate controls (A, C) and agrin KO hearts (B, D) labeling the coronary vasculature. Dorsal aspects are presented. Note the clearly defined and extended major vessels in the control heart (white arrows in A) which are virtually absent in the KO heart (white arrow in B). In E16.5 agrin KO heart, the main vessels are truncated (white arrows in D). (E-H), immunofluorescent staining on sections from E18.5 litter control hearts (E, G) and agrin KO (F, H) hearts for PECAM labeling endothelial cells. (G, H) are magnified views from the inset boxes in (E, F) showing a transverse section of a main coronary vessel. Note the weak and discontinuous PECAM staining in the agrin KO heart indicative of vessel instability (white arrow in H). Scale bars: A, B: 100 µm, C, D: 500 µm. E-H: 50µm.

## Figure S7

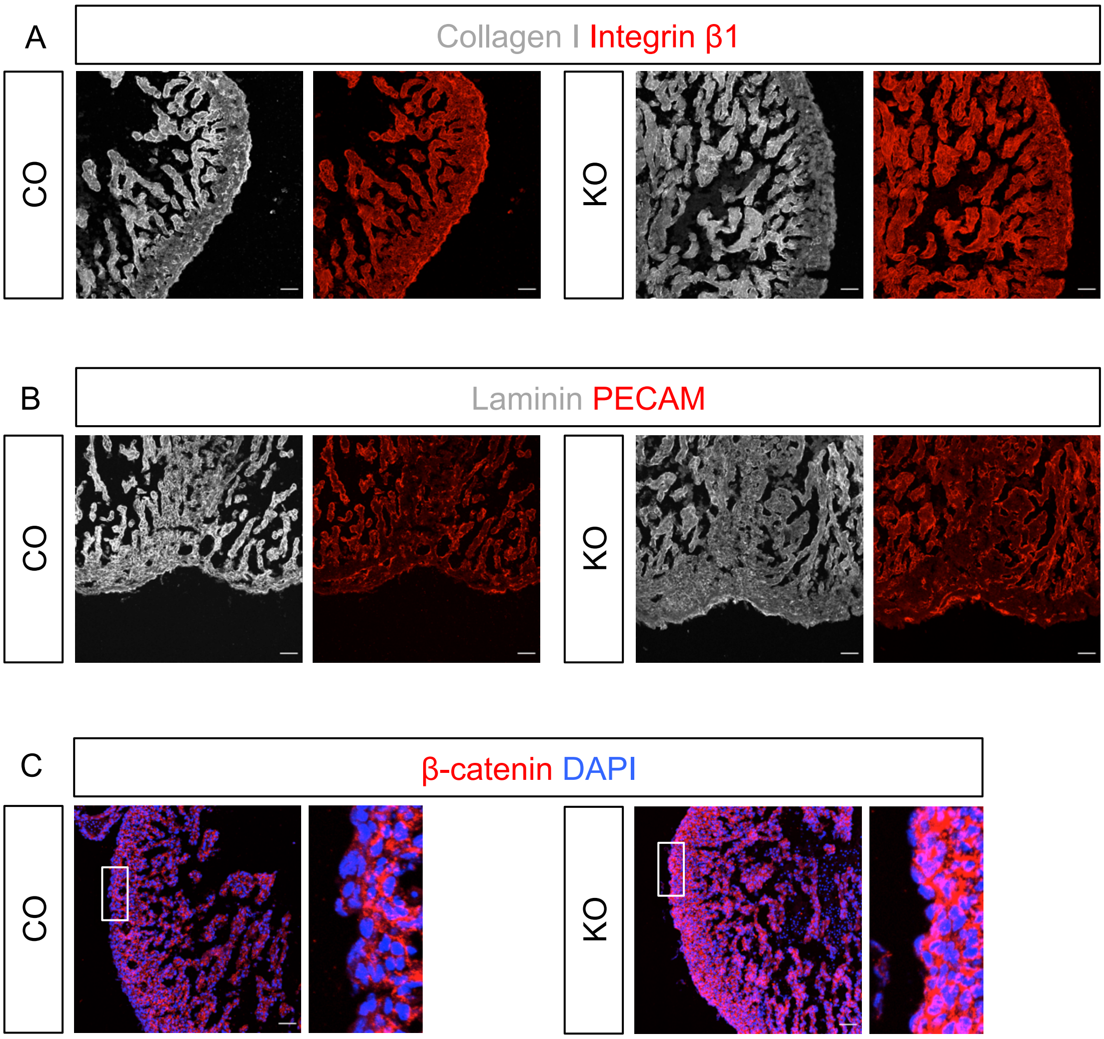


**Figure S7. ECM components are abnormally deposited in agrin KO myocardium.** Immunofluorescent staining of Collagen I (A, white), Integrin β1 (A, red), Laminin (B, white), PECAM (B, red) and β-catenin (C, red) in serial sections of embryonic hearts of E14.5 littermate control and agrin KO. Scale bars: 50 μm.

Development • Supplementary information

# Figure S8

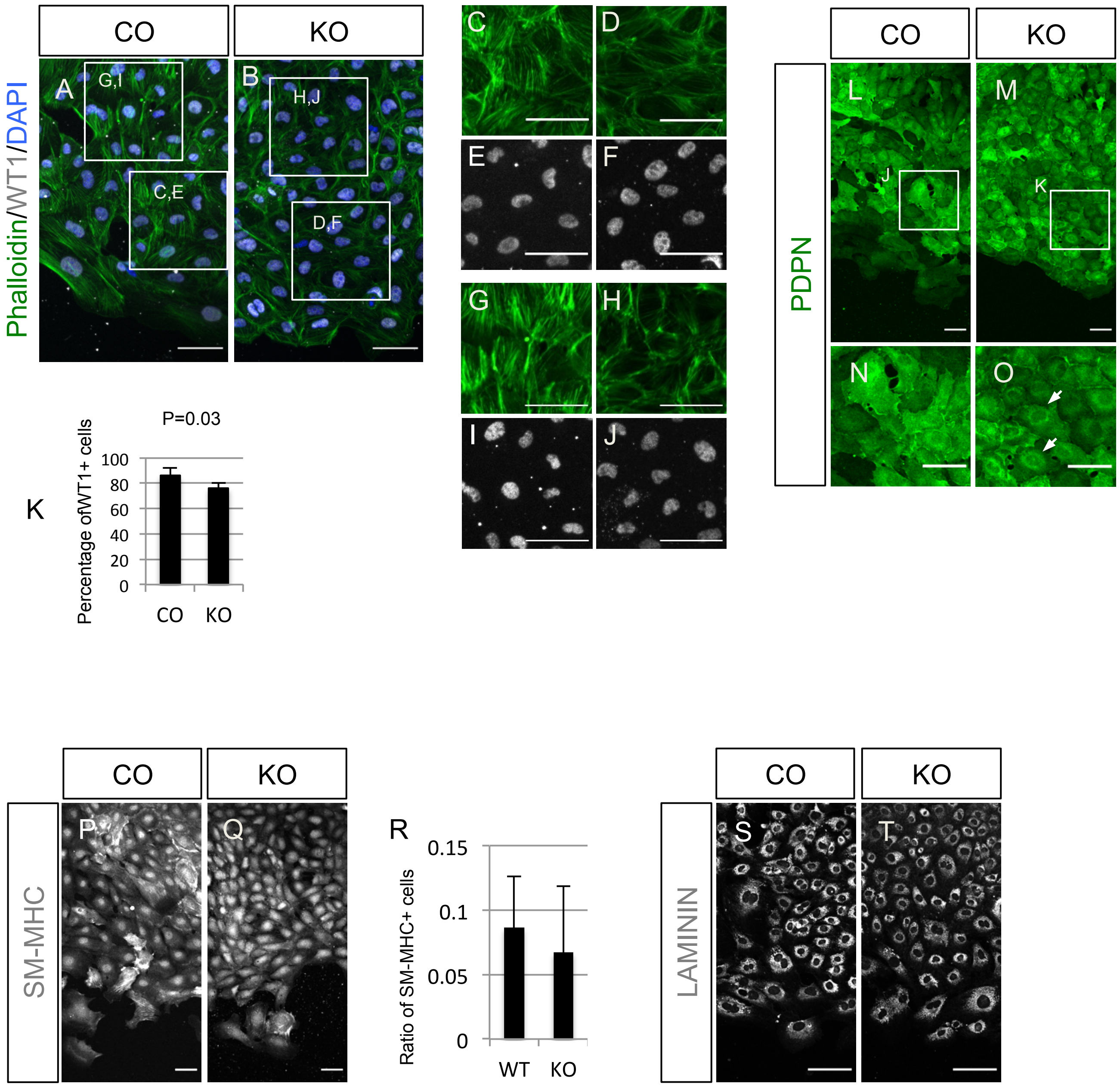
CO
KO
Phalloidin/WT1/DAPI
A
G,I
C,E
B
H,J
D,F
C
D
E
F
G
H
I
J
PDPN
L
M
N
O
K
P=0.03
Percentage ofWT1+ cells
100
80
60
40
20
0
CO
KO
SM-MHC
P
Q
R
Ratio of SM-MHC+ cells
0.15
0.1
0.05
0
WT
KO
LAMININ
S
T

**Figure S8. Migration and ECM defects in epicardium-derived cells from agrin-KO explants.**
(A-B) Immunofluorescent staining of epicardium-derived cells migrated away from E11.5 explants of littermate control and agrin KO for cell stress fibers (phalloidin, green), WT1 (white) and nuclei (DAPI). (C and E), (G and I) are magnified view of the inset boxes in (A). (D and F), (H and J) are magnified view of the inset boxes in (B). (K) Quantification of $WT1^+$ cells in control explant-derived and agrin KO derived epicardial cells. Data represent mean ± SEM. N=4 hearts per group. Significant differences (P-value) were calculated using an unpaired, two-tailed Student's *t*-test. (L, M) Immunofluorescent staining for podoplanin (green) in epicardium-derived cells from control and agrin KO explants. (N and O) are magnified view of the box in (L) and (M). Epicardial cells derived from the agrin KO explant showed weak podoplanin on the cell surface, especially in peri-nuclear regions (white arrows in O). (P, Q, S, T) Immunofluorescent staining for SM-MHC (P, Q) and Laminin (S, T) of epicardial cells from littermate control (P, S) and agrin KO explants (Q, T). (R): quantification of SM-MHC-positive cells in total explant-derived epicardial cells. Data represent mean ± S.E.M.; n=4 independent samples per group. Significant differences (P-value) were calculated using an unpaired, two-tailed Student's *t*-test. All scale bars: 50 µm.

# Figure S9

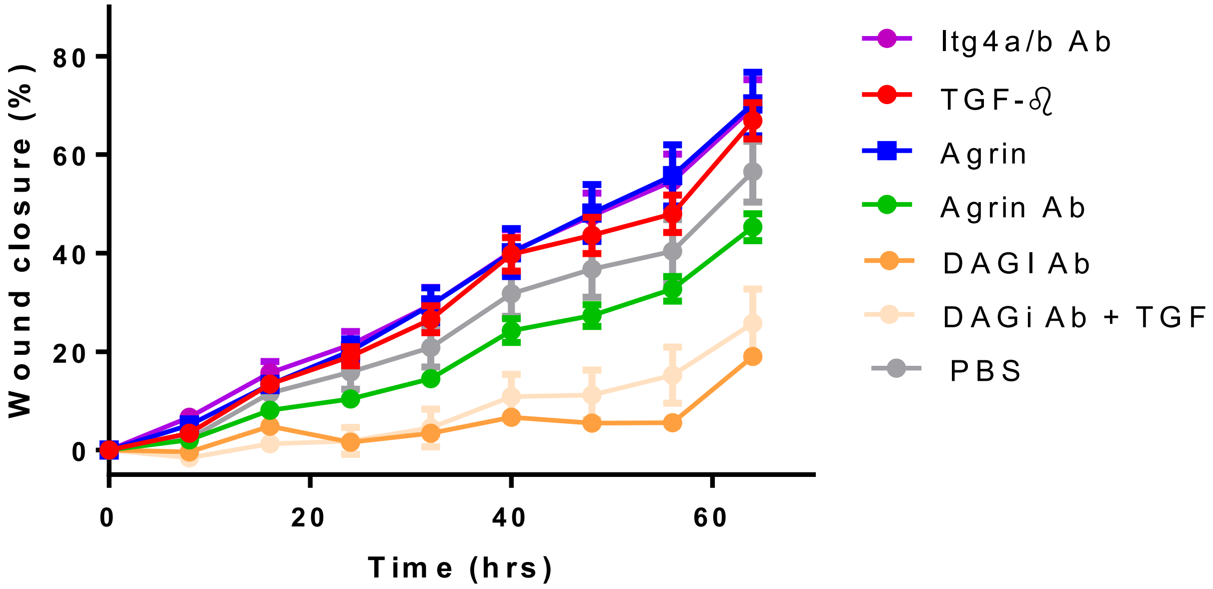


**Figure S9. Agrin enhances the migration of mouse epicardial cells.** Immortalised mouse epicardial cells were cultured for 64 hours with the indicated reagents and the wound closure was evaluated. Data represent mean ± SEM; n=12 independent treatments per group. Significant differences (P value) were calculated using one way ANOVA. Agrin treatment significantly increased the rate of 75%wound closure compared to untreated (PBS) and even TGFβ-treatment.

Development • Supplementary information

# Figure S10

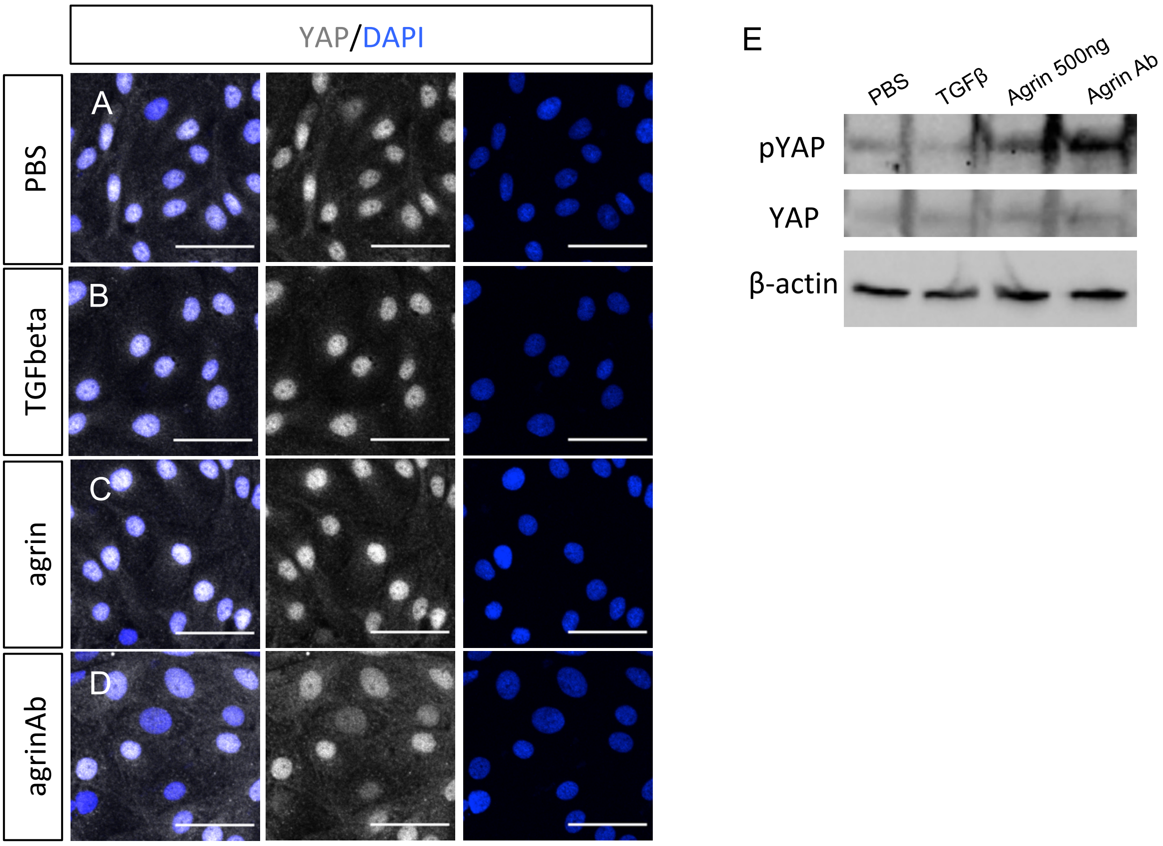


**Figure S10. Blocking agrin inhibits YAP in epicardial cells.** (A-D) Immunofluorescent staining of YAP (white) and DAPI (blue) in hESC-derived epicardial-like cells. (E) Western blot showing agrin and agrin antibody enhanced the expression of phosphorylated YAP. Scale bars: 10 µm.

**Table S1. Experimental models: organisms/strains**

| | | |
|---|---|---|
| Mouse: *Agrinfl/fl: B6.129-Agrn*$^{tm1Rwb}$*/J* | The Jackson Laboratory | JAX: 031788 |
| Mouse: *PGK-Cre*: *B6.C-Tg(Pgk1-cre)1Lni/CrsJ* | The Jackson Laboratory | JAX: 021160 |
| Mouse: *Wt1-CreERT2: WT1*$^{tm2(cre/ERT2)Wtp}$*/J* | The Jackson Laboratory | JAX: 010912 |
| Mouse: *Wt1-GFPCre: Wt1*$^{tm1(EGFP/cre)}$*Wtp/J* | The Jackson Laboratory | JAX: 010911 |

Development • Supplementary information

**Table S2. Antibodies**

| | | |
|---|---|---|
| Mouse monoclonal anti-human agrin Alexa647(D2) | Santa Cruz (1:100) | Cat# Sc374117-AF647 |
| Mouse monoclonal anti-agrin | EMD Milipore | Cat# MAB5204 |
| Rabbit polyclonal anti-mouse beta-catenin | Abcam (1:250) | Cat# AF2727 |
| Rabbit polyclonal anti-mouse collagen I | Abcam (1:200) | Cat# Ab34710 |
| Rat polyclonal anti-mouse CD31 (clone MEC13.3) | Abcam | Cat# 550274 |
| Goat monoclonal anti-mouse DAG1 | Abcam (1:200) | Cat# Ab136665 |
| Rat monoclonal anti-mouse EMCN (clone V.5C7) | Santa Cruz Biotech (1:50) | Cat# sc-53941 |
| Rabbit polyclonal anti-mouse fibronectin | Abcam (1:200) | Cat# 23750 |
| Rabbit polyclonal anti-human GM130 | Invitrogen (1:200) | Cat# 95727 |
| Rat monoclonal anti-mouse Integrin alpha4 | BD Pharmingen (1:200) | Cat# 553314 |
| Anti-integrin alpha4 (Natalizumab) | Absolute (1:100) | Cat# Ab00716-1.1 |
| Rat monoclonal anti-mouse integrin beta1 | EMD Millipore (1:200) | Cat# MAB1997 |
| Rabbit polyclonal anti-mouse total laminin | Abcam (1:200) | Cat# Ab11575 |
| Rabbit anti-mouse pFAK | Cell Signaling Technology (1:200) | Cat# 8556S |
| Rabbit polyclonal anti-mouse p-Histone H3 (Ser10) | Santa Cruz | Cat# Sc-56739 |
| Hamster monoclonal anti-mouse podoplanin (clone 103-M40) | Fitzgerald (1:200) | Cat# 10R-P155A |
| Rabbit polyclonal anti-mouse WT1 | Abcam(1:200) | Cat# Ab89901 |
| Rabbit polyclonal anti-mouse YAP | Abcam(1:200) | Cat# Ab89901 |
| Rabbit polyclonal anti-mouse YAP pY397 | Abcam(1:200) | Cat# Ab89901 |
| Rabbit polyclonal anti-mouse Zeb1 | Abcam(1:200) | Cat# Ab89901 |
| Rabbit polyclonal anti-mouse ZO1 | Abcam(1:200) | Cat# Ab89901 |
| Donkey anti-rat IgG Alexa Fluor 488 | Thermofisher | Cat# A21208 |
| Donkey anti-rat IgG Alexa Fluor 555 | Abcam | Cat# Ab150154 |
| Donkey anti-rabbit IgG Alexa Fluor 555 | Thermofisher | Cat# A32794 |

Development • Supplementary information

| | | |
|---|---|---|
| Donkey anti-rabbit IgG Alexa Fluor 647 | Thermofisher | Cat# A31573 |
| Goat anti-hamster IgG Alexa Fluor 488 | Thermofisher | Cat# A21110 |
| Donkey anti-goat IgG Alexa Fluor 488 | Thermofisher | Cat# A11055 |
| Donkey anti-goat IgG Alexa Fluor 555 | Abcam | Cat# Ab150134 |

Development • Supplementary information
