# Supplementary material for: The extracellular matrix protein agrin is essential for epicardial epithelial-to-mesenchymal transition during heart development
Source: Development. 2021 May 10;148(9):dev197525. doi: 10.1242/dev.197525 (PMC8172119; doi:10.1242/dev.197525)
Supplement: Reviewer comments [file develop-148-197525_review_history.pdf]

The extracellular matrix protein agrin is essential for epicardial epithelial-to-mesenchymal transition during heart development

Xin Sun, Sophia Malandraki-Miller, Tahnee Kennedy, Elad Bassat, Konstantinos Klaourakis, Jia Zhao, Elisabetta Gamen, Joaquim Miguel Vieira, Eldad Tzahor and Paul R. Riley
DOI: 10.1242/dev.197525

Editor: Benoit Bruneau

Review timeline

Original submission:	2 October 2020
Editorial decision:	16 November 2020
First revision received:	18 March 2021
Accepted:	3 April 2021

Original submission

First decision letter

MS ID#: DEVELOP/2020/197525

MS TITLE: The extracellular matrix protein agrin is essential for epicardial epithelial-to-mesenchymal transition during heart development

AUTHORS: Xin Sun, Sophia Malandraki-Miller, Tahnee Kennedy, Elad Bassat, Konstantinos Klaourakis, Jia Zhao, Elisabetta Gamen, Joaquim Vieira, Eldad Tzahor, and Paul Riley

I have now received all the referees' reports on the above manuscript, and have reached a decision. The referees' comments are appended below, or you can access them online: please go to BenchPress and click on the 'Manuscripts with Decisions' queue in the Author Area.

As you will see, the referees express considerable interest in your work, but have some significant criticisms and recommend a revision of your manuscript before we can consider publication. If you are able to revise the manuscript along the lines suggested, which may involve further experiments, I will be happy receive a revised version of the manuscript. Your revised paper will be re-reviewed by one or more of the original referees, and acceptance of your manuscript will depend on your addressing satisfactorily the reviewers' major concerns. Please also note that Development will normally permit only one round of major revision.

We are aware that you may be experiencing disruption to the normal running of your lab that make experimental revisions challenging. If it would be helpful, we encourage you to contact us to discuss your revision in greater detail. Please send us a point-by-point response indicating where you are able to address concerns raised (either experimentally or by changes to the text) and where you will not be able to do so within the normal timeframe of a revision. We will then provide further guidance. Please also note that we are happy to extend revision timeframes as necessary.

Please attend to all of the reviewers' comments and ensure that you clearly highlight all changes made in the revised manuscript. Please avoid using 'Tracked changes' in Word files as these are lost in PDF conversion. I should be grateful if you would also provide a point-by-point response detailing how you have dealt with the points raised by the reviewers in the 'Response to Reviewers' box. If you do not agree with any of their criticisms or suggestions please explain clearly why this is so.

© 2021. Published by The Company of Biologists under the terms of the Creative Commons Attribution License (https://creativecommons.org/licenses/by/4.0/).

Reviewer 1

Advance summary and potential significance to field

In their article, Sun et al. shed new light on the role of the ECM component agrin on epicardial epithelial to mesenchymal transition during cardiac development. They demonstrate the importance of the ECM on the cytoskeletal connectivity and mechanical integrity of the epicardial cells using robust assays and convincing microscopy imaging. Agrin had been reported to regulate epithelial-mesenchymal transition through focal adhesion integrity and its role has been described in the filed of cancer biology and heart repair/regeneration. This manuscript highlight for the first time a role in agrin in heart development and is of will be of great interest to developmental biologists.

Comments for the author

Sun et al. have written a very detailed and thoroughly conducted study, the presented data is well articulated and convincing.

The manuscript would benefit from an extended supplemental version of the Material and method sections with clear details as most experiments would not be reproducible by other researchers with the information provided at present.

The authors cited the work of Chakraborty (2017, 2018) which linked agrin to the Hippo signalling pathway. Since Hippo signalling has been shown to be a key player in epicardial to mesenchymal transition (Singh at al, 2016, Cell reports), have the authors investigated if it was affected in their in vivo and in vitro models? These additional experiments could potentially shed some light into a molecular mechanism downstream of agrin and its receptor Dystroglycan.

Additional suggestion: Fig 7K-7O: Could this figure be expanded to a comparison of GM130 and DAG1 between Control and agrin-/- mice hearts?

Minor comments:
- Referencing:

Check alphabetical order of bibliography (Gise before Gautam)

Baehr et al 2020 (not 2019 as written in bibliography).

There are many references in the text that are missing in the bibliography:
Balmer et al. 2014, Kwee et al 1995, Riley 2012, Von Gise et al 2011, Vega-Hernandez et al 2011, Compton et al 2007, Zhou et al 2012, Smart et al 2007 Niktin et al 1987, Lin et al 2001, Pankov and Yamada 2002, Ervasti and Campbell 1993, Mitra et al 2005… Please check all references thoroughly.

Line 115: Basset et al 2017, should be Bassat et al 2017. You could also mention this recent review relevant to heart regeneration: Bigotti et al 2020.

- Other minor comments:

Line 62: word missing between “contribute” and “cells”
Line 82: space missing before However.
Line 86: “the” missing between “of” and “venous”
Line 214: Should be Figures 2H, 2L.
Line 215: Should be Figures 2I, 2M.
Figure 4R frame U, W (should be T,V?) and 4S frame V, X (should be U,W?).
Pattern and intensity of signal for PDPN (Frame in Figure 4S) is not correlated with signal/pattern in 4U.
Figure 4I, N, O, X: How the percentage is calculated? Was the whole section analysed? How many serial sections of N hearts were analysed?
Fig 5I, 5J: Very difficult to interpret because the beta catenin signal seems to be oversaturated.

© 2021. Published by The Company of Biologists under the terms of the Creative Commons Attribution License (https://creativecommons.org/licenses/by/4.0/).

Fig 5K, 5L: How many serial sections of 3 hearts? Were the staining experiments all done at the same time to avoid inter-experimental variations? Or was the IF signal normalised between experiments?
Fig S5A-D: Please provide WT1 staining on its own (without DAPI) as WT1 staining is very difficult to visualise.
Fig S5E: Graph needs Y axis label.
Gig S5D: One could argue that the chosen area is not representative as frame D has been placed exactly where the lowest phalloidin signal can be found.
Cells at the edge in Control explant seem to be larger, if this is quantifiable and a significant difference found, it would reinforce the hypothesis. This is also clear on Fig S5F-J. Cells are more compacted at the edge of the explant in KO explants.
P Values for Fig6 and FigS6 should not be calculated with a Student's t-test. Please use adequate statistical analysis.

Reviewer 2

Advance summary and potential significance to field

This manuscript performs an analysis of the epicardium of the developing heart in wild type and agrin mutant mice. They conclude using immunohistochemistry that there are key components of the ECM where there are high and low levels of epicardial EMT. One component localized to active EMT regions is agrin. Agrin deficiency compromised epicardial development in hearts. Analysis of the effect of agrin on ESC-derived epicardial cells revealed that it causes a decrease in junctional b-catenin and promoted pFAK at focal adhesions. It also caused aggregation of dystroglycan at the golgi. These results provide a further understanding of the role of this critical ECM protein that has been shown to stimulate heart regeneration.

Comments for the author

Major comments

1. There is a lack of evidence for EMT in most cases presented. The authors should provide additional parameters before they identify cells as undergoing EMT. They show morphology, downregulation of WT1, and loss of PDPN junctions.
There should be more definitive evidence as accepted by the field such as induction of EMT genes (slug/snail), evidence of leaving the epicardial layer and moving into the subepicardial space, etc.
2. Antibodies for integrins and ECM proteins in Fig. 2 and Fig. 3 do not look like they have specific signal and this reviewer would not come to the same conclusions and the authors based on the images shown. The authors should validate that the signal they claim to observe is specific. One option is to add a control tissue where they are known positive and negative regions and the antibody signal is specific to the expected regions. Agrin should be validated by staining knockout hearts.
3. All the effects for which just an image is shown should be quantified and statistics performed.
4. The PH3 staining looks non-specific in Fig. 4. Furthermore, having 30% of cells positive is much higher than expected. Since PH3 only stains during certain short parts of the cell cycle it is rarer than 30%.
5. In knockout hearts where there is a decrease shown for certain proteins an internal control antibody that does not change on that exact tissue must also be shown to confirm that decreased fluorescence is not due to overall tissue degradation or differential handling of that sample during immunofluorescent labeling. Better is to validate differences using western blot.
6. The immunofluorescence in Fig 5K and L require validation such as western blotting since the labeling is non-specific.
7. Figs 6 and 7, images should be accompanied with quantification from a statistically appropriate number of experiments and images.
8. In fig 7H-J, this reviewer can see areas in the control images where DAG1 and GM130 overlap, although authors state that it does not. Needs quantification.

© 2021. Published by The Company of Biologists under the terms of the Creative Commons Attribution License (https://creativecommons.org/licenses/by/4.0/).

Reviewer 3

Advance summary and potential significance to field

The manuscript by Sun et al. report a functional analysis of the ECM protein agrin in the regulation of EMT in the embryonic epicardium. The authors report first a morphological analysis of EMT in the embryonic heart. They describe a focal presence of EMT, which produces islands of mesenchymal cells adjacent to epicardial cells maintaining an epithelial state. Using antibodies to different matrix proteins a detailed high-resolution analysis of the modulation of expression during EMT in the epicardium is provided. The authors then analysed the phenotype of a global agrin knockout and demonstrate impaired epicardial development, reduction in EMT and a lack of myocardial invasion of EPDCs and an impaired coronary artery development. Using epicardial explant cultures, the distribution of various epicardial marker proteins were investigated. Using epicardial cells differentiated from human iPSCs, the effects of agrin on EMT were investigated and compared to TGFbeta. Interestingly, agrin modulates the subcellular localisation of Dystroglycan, which accumulates in the Golgi apparatus in response to agrin treatment in cultured epicardial cells and in the embryonic heart.
This a very nice and carefully executed study. I have only a few remarks.

Comments for the author

1. Why the authors refer to the scanning electron microscopy study in Figure 1 as being a study at subcellular resolution. I think SEM informs about the surface morphology but does not provide subcellular resolution?
2. Dystroglycan-agrin interaction and the effect of agrin treatment on dystroglycan accumulation in the Golgi apparatus. While effects of dystroglycan missense mutations in skeletal muscle have been demonstrated, I am not aware of any study linking dystroglycan mutations either as null or knockin mutation in the mouse or in patients to impaired epicardialisation. Likewise, numerous proteins involved in glycosylation of dystroglycan have been identified to cause limb-girdle muscular dystrophy, but I am not aware of any association of these glycosyltransferases with epicardium formation. It would be nice to address this question in further detail in the discussion.

Minor:
Line 146 tightly connected
Line 186 distribution of key ECM components and integrins
Line 196 These proteins were all detected…
Line 292 significantly downregulated
Line 308 add proper legend for the X-axis in Fig. 5SE
Line 337 In addition to stress fiber density, we assessed the levels of phosphorylation of focal adhesion kinase (FAK) at tyrosine 397 (pFAK Y397), which connects the extracellular matrix signals and intracellular signaling through integrins and is a key regulator of cell migration, an important process in cells undergoing EMT (reviewed by Mitravet al., 2005, Larsen et al., 2006). Focal adhesion kinase (FAK) is a tyrosine kinase connecting extracellular matrix signals and intracellular signaling through integrin. It is a key regulator for cell migration. P hosphorylation of FAK at Tyrosine 397 (pFAK Y397) results its recruitment to focal adhesion nd serving as signaling axis (reviewed by Mitra et al., 2005, Larsen et al., 2006).
Duplication. Remove one or the other sentence.
Line 368 … human epicardium-like cells following exogenous agrin treatment
Line 459 they have been described
Line 487 neuromuscular junction
Line 513 dystroglycan

© 2021. Published by The Company of Biologists under the terms of the Creative Commons Attribution License (https://creativecommons.org/licenses/by/4.0/).

First revision

Author response to reviewers' comments

Reviewer 1:
In their article, Sun et al. shed new light on the role of the ECM component agrin on epicardial epithelial to mesenchymal transition during cardiac development. They demonstrate the importance of the ECM on the cytoskeletal connectivity and mechanical integrity of the epicardial cells using robust assays and convincing microscopy imaging. Agrin had been reported to regulate epithelial-mesenchymal transition through focal adhesion integrity and its role has been described in the field of cancer biology and heart repair/regeneration. This manuscript highlights for the first time a role inagrin in heart development and is of will be of great interest to developmental biologists.

Reviewer 1 Comments for the Author: Sun et al. have written a very detailed and thoroughly conducted study, the presented data is well articulated and convincing. The manuscript would benefit from an extended supplemental version of the Material and method sections with clear details as most experiments would not be reproducible by other researchers with the information provided at present.

We would like to thank the reviewer for the positive assessment of our study and suggestions for improvement. The Materials and methods section has now been improved with more details and clearer descriptions.

The authors cited the work of Chakraborty (2017, 2018) which linked agrin to the Hippo signalling pathway. Since Hippo signalling has been shown to be a key player in epicardial to mesenchymal transition (Singh at al, 2016, Cell reports), have the authors investigated if it was affected in their in vivo and in vitro models? These additional experiments could potentially shed some light into a molecular mechanism downstream of agrin and its receptor, Dystroglycan.

We would like to thank the reviewer for this suggestion. We have now investigated the effect of agrin on YAP in hEPDCs. The results are included in a new Supplementary Figure 10 and discussed on page 14, lines 392-410 of the revised manuscript.

Additional suggestion: Fig 7K-7O: Could this figure be expanded to a comparison of GM130 and DAG1 between Control and agrin-/- mice hearts?

We have incorporated a comparison of GM130 and DAG1 in E14.5 agrin mutants into Figure 7. The result is discussed on page 15, lines 444-449 of the revised manuscript.

Minor comments:
•Referencing:
Check alphabetical order of bibliography (Gise before Gautam) Baehr et al 2020 (not 2019 as written in bibliography).

There are many references in the text that are missing in the bibliography: Balmer et al. 2014, Kwee et al 1995, Riley 2012, Von Gise et al 2011, Vega- Hernandez et al 2011, Compton et al 2007, Zhou et al 2012, Smart et al 2007, Niktin et al 1987, Lin et al 2001, Pankov and Yamada 2002, Ervasti and Campbell 1993, Mitra et al 2005… Please check all references thoroughly.

Line 115: Basset et al 2017, should be Bassat et al 2017. You could also mention this recent review relevant to heart regeneration: Bigotti et al 2020.

We apologize for the omission, and any errors, in the citing of references; these mistakes have now been corrected.

•Other minor comments:
Line 62: word missing between "contribute" and "cells" Line 82: space missing before However.

© 2021. Published by The Company of Biologists under the terms of the Creative Commons Attribution License (https://creativecommons.org/licenses/by/4.0/).

Line 86: "the" missing between "of" and "venous" Line 214: Should be Figures 2H, 2L.

Line 215: Should be Figures 2I, 2M.

We apologise for these typos all of which have now been corrected in the revised manuscript.

Figure 4R frame U, W (should be T,V?) and 4S frame V, X (should be U,W?).

Pattern and intensity of signal for PDPN (Frame in Figure 4S) is not correlated with signal/pattern in 4U.

The frame in Figure 4S is now moved to the correct place representing 4U.

Figure 4I, N, O, X: How the percentage is calculated? Was the whole section analysed? How many serial sections of N hearts were analysed?

We now have removed the proliferation quantification due to not being able to collect further agrin mutant embryos because of recent limited access to our biological services facility and animal breeding issues. This does not compromise our findings on agrin's role in epicardial EMT. Instead we include quantification of WT1+ nuclei in the epicardium and myocardium as indicating EMT and as presented in the revised Figure 4I, and 4J. WT1+ nuclei in epicardium and total nuclei (labelled with DAPI and podoplanin) were counted respectively, and the ratio was calculated and compared between the control group (N=3) and KO group (N=3). For WT1+ cells in the myocardium (Figure 4O), WT1+ cells were manually counted in defined regions and the total DAPI positive area per region (intensity 50-255) was measured with ImageJ. WT1+ nuclei relative to DAPI was calculated and compared between the control group (N=3) and KO group (N=3). For Figure 4I and 4J, 2-4 regions from a section were cropped out for quantification. 2-3 serial sections were analysed for each sample. For WT1+ cell quantification presented in the revised Figure 4S, a region on the surface of an E16.5 embryonic heart was selected and WT1+ nuclei as well as total nuclei were manually counted. The ratio of WT1+ nuclei number to total nuclei number was calculated and compared between the control group (N=3) and KO group (N=3). From each heart, 2 regions from similar locations were selected for quantification. The details on quantification and sample numbers, are now included in the revised Material and Methods page 22, lines 613- 624.

Fig 5I, 5J: Very difficult to interpret because the beta catenin signal seems to be oversaturated.

We have now included additional β-catenin staining in Supplementary Figure 7C. These new data in combination with the immunostaining data in Figure 5I and 5J clearly show the up-regulation of β-catenin in agrin KOs compared with littermate controls.

Fig 5K, 5L: How many serial sections of 3 hearts? Were the staining experiments all done at the same time to avoid inter-experimental variations? Or was the IF signal normalised between experiments?

For each sample (3 controls and 3 KOs), 2-4 serial sections were imaged at both left and right ventricular walls as well as the interventricular septum. The staining of the same set of markers were all performed and scanned at the same time to avoid experimental variations. We have also included additional staining of collagen I and laminin on different sections in Supplementary Figure 7A and 7B to demonstrate the antibody specificity. We further present integrin β1 and PECAM staining on the same sections, with collagen I and laminin, respectively, and the intensity of integrin β1 and PECAM is comparable between the control and the KO sections, indicating consistent staining across samples. The detailed quantification methodology is now explained in the revised Material and Methods, page 22 lines 626-630.

Fig S5A-D: Please provide WT1 staining on its own (without DAPI) as WT1 staining is very difficult to visualise.

The WT1 only channel is now provided in the revised new Supplementary figure 8C-8J.

Fig S5E: Graph needs Y axis label.

© 2021. Published by The Company of Biologists under the terms of the Creative Commons Attribution License (https://creativecommons.org/licenses/by/4.0/).

This has been corrected in the revised Supplementary Figure 8K. The Y axis is the percentage of WT1+ cells.

Fig S5D: One could argue that the chosen area is not representative as frame D has been placed exactly where the lowest phalloidin signal can be found.

Cells at the edge in Control explant seem to be larger, if this is quantifiable and a significant difference found, it would reinforce the hypothesis. This is also clear on Fig S5F-J. Cells are more compacted at the edge of the explant in KO explants.

We now provide more images of phalloidin of both the leading edge and central area of the explants to support our conclusion in Supplementary Figure 8A-8J. Although we also noted that loss of agrin results in apparently “smaller” leading edge cells, both cell shapes and size can vary between explants, even of the same genotype, and so we have refrained from drawing any further conclusions on cell size at this juncture.

P Values for Fig6 and FigS6 should not be calculated with a Student’s t- test. Please use adequate statistical analysis.

We apologize for this oversight and the correct use of one-way ANOVA has been incorporated in the revised manuscript, page 23, lines 632-636 and Supplementary Figure 10 legends.

Reviewer 2:

Advance Summary and Potential Significance to Field:
This manuscript performs an analysis of the epicardium of the developing heart in wild type and agrin mutant mice. They conclude using immunohistochemistry that there are key components of the ECM where there are high and low levels of epicardial EMT. One component localized to active EMT regions is agrin. Agrin deficiency compromised epicardial development in hearts. Analysis of the effect of agrin on ESC-derived epicardial cells revealed that it causes a decrease in junctional b-catenin and promoted pFAK at focal adhesions. It also caused aggregation of dystroglycan at the golgi. These results provide a further understanding of the role of this critical ECM protein that has been shown to stimulate heart regeneration.

We thank the reviewer for the assessment of our study, constructive criticism and suggested experiments to improve the manuscript.

Major comments.
1. There is a lack of evidence for EMT in most cases presented. The authors should provide additional parameters before they identify cells as undergoing EMT. They show morphology, downregulation of WT1, and loss of PDPN junctions. There should be more definitive evidence as accepted by the field such as induction of EMT genes (slug/snail), evidence of leaving the epicardial layer and moving into the subepicardial space, etc.

Snail1 is expressed in embryonic epicardium, but not Snail2. Moreover, Snail1 is not required for epicardial EMT (Casanova et al., 2013, Genesis 51:32-40, https://doi.org/10.1002/dvg.22353). We investigated other well-characterized EMT markers such as ZEB1 and ZO1 in the E13.5 and E14.5 epicardium. The data are included in Supplementary Figure 3. Our new data shows that epicardial cells express ZEB1 and ZO1 in a heterogeneous manner, consistent with our results presented in Figure 2. We would also suggest that the morphology of the cells and their attachment status on the basal membrane (as labeled with laminin in Figure 2) are also proof of EMT, as reviewed in Yang et al., (2020), *Nat Rev Mol Cell Bio* 1-12. https://doi.org/10.1038/s41580-020-0237-9
.

2. Antibodies for integrins and ECM proteins in Fig. 2 and Fig. 3 do not look like they have specific signal and this reviewer would not come to the same conclusions and the authors based on the images shown. The authors should validate that the signal they claim to observe is specific. One option is to add a control tissue where they are known positive and negative regions and the antibody signal is specific to the expected regions. Agrin should be validated by staining knockout

© 2021. Published by The Company of Biologists under the terms of the Creative Commons Attribution License (https://creativecommons.org/licenses/by/4.0/).

hearts.

We respectfully disagree that the antibody stainings presented are non-specific. The antibodies used for Collagen I, laminin, integrin α4, podoplanin, integrin β1, fibronectin, β- catenin (Catalogue numbers listed in Material and Methods) have been validated in many published studies to-date. That said, to address this issue more directly we have added integrin α4 staining at E10.5 in Figure 2 to show its specificity to epicardium before the initiation of epicardial cell EMT. Moreover, we also provide positive controls for Integrin α4 and laminin. These results are presented in Supplementary Figure 5. Our data shows that integrin β1 and fibronectin broadly express in embryonic hearts and do not correlate with *high- or low-EMT region, in contrast to integrin α4 and laminin. The epicardium and cardiac lymphatics are the well-recognized positive control tissue-types for podoplanin, as indicated in our wholemount staining (Figure 1 and Figure 4).*

The agrin constitutive deletion has been previously published. Validation of different agrin knockout alleles has been presented in Gautam et al., (1996) *Cell* **85**, 525–535 and Harvey et al., (2007) *Am J Pathol* **171**, 139–152; we have cited these studies on page 10 line 260- 265.

3. All the effects for which just an image is shown should be quantified and statistics performed.

Our data in Figure 2, Figure 4, Figure 5, Figure 6 and Figure 7 focuses on the ECM pattern and localization changes, rather than expression level changes- therefore, this does not require quantification. Rather we have presented quantification data for the relevant findings in Figure 4 and Figure 5 where we draw references to expression level changes and changes in numbers of positive cells.

4. The PH3 staining looks non-specific in Fig. 4. Furthermore, having 30% of cells positive is much higher than expected. Since PH3 only stains during certain short parts of the cell cycle it is rarer than 30%.

We have removed the proliferation of epicardial cells from Figure 4 since we were not able to validate this finding in further mutant embryos due to limited (lock-down) access to our animal facility and resultant animal breeding difficulties. Removal of this data does not affect our overall conclusion that agrin is important for epicardial EMT.

5. In knockout hearts where there is a decrease shown for certain proteins, an internal control antibody that does not change on that exact tissue must also be shown to confirm that decreased fluorescence is not due to overall tissue degradation or differential handling of that sample during immunofluorescent labeling. Better is to validate differences using western blot.

For each group of experiments, all the samples were processed, stained and scanned at the *same time. The experiment procedure is now further explained on page 22, lines 626-630. The agrinKO and the control showed similar expression level of integrin β1, as presented in Figure 5C and 5D, indicating the tissues are intact and handled consistently between experiments. Staining of integrin β1 in Figure 5C and 5D is from the same experiment with Collagen I (Figure 5E and 5F), serving as an internal control as requested. In addition, we now provide co-staining images of collagen I with integrin β1, as well as laminin and CD31. These results are presented in Supplementary figure 7A and 7B to show the specific down- regulation of Collagen I and Laminin, but not integrin β1 and CD31, in the epicardium and myocardium of KOs versus control sections. The use of multiple antibodies in combination here, where specific expression changes are observed in some but not all markers, serves as an important control for comparable tissue integrity and equivalent sample/section processing.*

6. The immunofluorescence in Fig 5K and L require validation such as western blotting since the labeling is non-specific.

We have provided immunostaining of Collagen I and Laminin within the ventricular wall to demonstrate the specificity of these antibodies. These data are presented in Supplementary Figure 7A and 7B and discussed in revised manuscript on page 11, lines 298-305.

© 2021. Published by The Company of Biologists under the terms of the Creative Commons Attribution License (https://creativecommons.org/licenses/by/4.0/).

7. Figs 6 and 7, images should be accompanied with quantification from a statistically appropriate number of experiments and images.

These figures are highlighting spatial differences in localization not changes in overall expression, so quantification is not informative. In Figure 6, our results clearly reveal enhanced pFAK localization on focal adhesions and since we do not compare the EMT effect between different treatments for this staining, we do not think quantification of pFAK here is necessary to support our hypothesis. Similarly, the ß-catenin staining shows clear decreased localization at cell-cell borders, but not necessarily changes in total ß-catenin. Dystroglycan localization (Figure 7) is discussed in under point 8) below.

8. In fig 7H-J, this reviewer can see areas in the control images where DAG1 and GM130 overlap, although authors state that it does not. Needs quantification.

We apologise for any confusion here and have revised this part in manuscript on page 15 lines 428-439. Our result clearly shows that agrin enhanced localization of Dystroglycan in Golgi apparatus as well as in the nuclear envelope. Without agrin treatment, Dystroglycan can still be detected in the nuclear envelope and the connecting Golgi apparatus, as pointed out by this reviewer. We respectfully disagree that quantification is needed to further address this, as this is again illustrative of an agrin-induced change in spatial localization, rather than a quantified expression change; the enhanced localization of Dystroglycan within the Golgi apparatus by agrin is apparent in Figure 7.

Reviewer 3:

Advance Summary and Potential Significance to Field:

The manuscript by Sun et al. report a functional analysis of the ECM protein agrin in the regulation of EMT in the embryonic epicardium. The authors report first a morphological analysis of EMT in the embryonic heart. They describe a focal presence of EMT, which produces islands of mesenchymal cells adjacent to epicardial cells maintaining an epithelial state. Using antibodies to different matrix proteins a detailed high-resolution analysis of the modulation of expression during EMT in the epicardium is provided. The authors then analysed the phenotype of a global agrin knockout and demonstrate impaired epicardial development, reduction in EMT and a lack of myocardial invasion of EPDCs and an impaired coronary artery development. Using epicardial explant cultures, the distribution of various epicardial marker proteins were investigated. Using epicardial cells differentiated from human iPSCs, the effects of agrin on EMT were investigated and compared to TGFbeta. Interestingly, agrin modulates the subcellular localisation of Dystroglycan, which accumulates in the Golgi apparatus in response to agrin treatment in cultured epicardial cells and in the embryonic heart. This a very nice and carefully executed study. I have only a few remarks.

We would like to thank this reviewer for this very positive assessment.

1. Why the authors refer to the scanning electron microscopy study in Figure 1 as being a study at subcellular resolution. I think SEM informs about the surface morphology but does not provide subcellular resolution?

We have corrected this statement on subcellular resolution and refer to use of SEM for high resolution of the topography of the outer apical surface of the epicardial layer. This has been corrected in the abstract on page 1, line 36 and on page 16, lines 468-476.

2. Dystroglycan-agrin interaction and the effect of agrin treatment on dystroglycan accumulation in the Golgi apparatus. While effects of dystroglycan missense mutations in skeletal muscle have been demonstrated, I am not aware of any study linking dystroglycan mutations either as null or knockin mutation in the mouse or in patients to impaired epicardialisation. Likewise, numerous proteins involved in glycosylation of dystroglycan have been identified to cause limb-girdle muscular dystrophy, but I am not aware of any association of these glycosyl transferases with epicardium formation. It would be nice to address this question in further detail in the discussion.

We would like to thank the reviewer for this suggestion. The discussion on dystroglycan has now

© 2021. Published by The Company of Biologists under the terms of the Creative Commons Attribution License (https://creativecommons.org/licenses/by/4.0/).

been revised in the manuscript pages 18-19, lines 530-558. Although dystroglycan has been well characterized for its role in basal membrane assembly at the cellular and subcellular level, and as applied to muscular dystrophy in terms of muscle structure and regeneration, the impact of dystroglycan in heart development remains unknown. One possibility is that dystroglycan loss in the epicardium, may result in impaired assembly of the forming epicardial basal membrane which may impact on downstream processes such as myocardial trabeculation, ventricle wall compaction or coronary vessel formation resulting in early embryo death.

Minor:
Line 146 tightly connected

Line 186 distribution of key ECM components and integrins
Line 196 These proteins were all detected…
Line 292 significantly downregulated

Line 308 add proper legend for the X-axis in Fig. 5SE

Line 337 In addition to stress fiber density, we assessed the levels of phosphorylation of focal adhesionkinase (FAK) at tyrosine 397 (pFAK Y397), which connects the extracellular matrix signals and intracellularsignaling through integrins and is a key regulator of cell, an important process in cellsundergoing EMT (reviewed by Mitravet al., 2005, Larsen et al., 2006). Focal adhesion kinase (FAK) is a tyrosine kinase connecting extracellular matrix signals and intracellular signaling through integrin. It is akey regulator for cell migration. P hosphorylation of FAK at Tyrosine 397 (pFAK Y397) results its recruitment to focal adhesion and serving as signaling axis (reviewed by Mitra et al., 2005, Larsen et al., 2006). Duplication. Remove one or the other sentence.

Line 368… human epicardium-like cells following exogenous agrin treatment

Line 459 they have been described

Line 487 neuromuscular junction

Line 513 dystroglycan

We apologise for these typos, all of which have now been corrected in the revised manuscript.

Second decision letter

MS ID#: DEVELOP/2020/197525

MS TITLE: The extracellular matrix protein agrin is essential for epicardial epithelial-to-mesenchymal transition during heart development

AUTHORS: Xin Sun, Sophia Malandraki-Miller, Tahnee Kennedy, Elad Bassat, Konstantinos Klaourakis, Jia Zhao, Elisabetta Gamen, Joaquim Vieira, Eldad Tzahor, and Paul Riley
ARTICLE TYPE: Research Article

I am happy to tell you that your manuscript has been accepted for publication in Development, pending our standard ethics checks.

© 2021. Published by The Company of Biologists under the terms of the Creative Commons Attribution License (https://creativecommons.org/licenses/by/4.0/).

Reviewer 1

Advance summary and potential significance to field

In their article, Sun et al. shed new light on the role of the ECM component agrin on epicardial epithelial to mesenchymal transition during cardiac development. They demonstrate the importance of the ECM on the cytoskeletal connectivity and mechanical integrity of the epicardial cells using robust assays and convincing microscopy imaging. Agrin had been reported to regulate epithelial-mesenchymal transition through focal adhesion integrity and its role has been described in the field of cancer biology and heart repair/regeneration. This manuscript highlights for the first time a role in agrin in heart development and will be of great interest to developmental biologists.

Comments for the author

I thank the authors for their thorough and detailed reply. My comments have been addressed satisfactorily.

Reviewer 2

Advance summary and potential significance to field

This study is a description of the Agrin knockout phenotype, which is an ECM protein that stimulates heart regeneration.

Comments for the author

From the rebuttal, it appears that the authors and I fundamentally disagree about what is required of data to be reported. I believe that data must always be quantified and statistics performed to demonstrate that an effect was seen in more than the one image that is shown in a Figure. A lack of quantification was my main comment to the authors. The authors replied to most of my these that quantification was either not informative or not required.

© 2021. Published by The Company of Biologists under the terms of the Creative Commons Attribution License (https://creativecommons.org/licenses/by/4.0/).
